# Structural Vulnerability in Health Research: A Systematic Mixed Studies Review

**DOI:** 10.1111/jan.70408

**Published:** 2025-12-01

**Authors:** Levia A. Sutton, Carmen Alvarez, Therese S. Richmond, Sara F. Jacoby

**Affiliations:** ^1^ University of Pennsylvania School of Nursing Philadelphia Pennsylvania USA

**Keywords:** health equity, nursing, parallel‐results convergent synthesis, structural determinants of health, structural vulnerability, systematic mixed studies review

## Abstract

**Aims:**

To systematically examine how structural vulnerability has been defined and operationalised in United States‐based health research, identify conceptual consistencies and methodological gaps, and propose core dimensions of structural vulnerability along with implications for future application in health research.

**Design:**

A systematic mixed‐studies review using a parallel‐results convergent synthesis design.

**Data Sources:**

PubMed, Embase, Scopus and CINAHL were searched from first publication through 2024 using the terms ‘structural* vulnerab*’ AND health.

**Review Methods:**

Peer‐reviewed English‐language empirical studies conducted in the United States that applied the concept of structural vulnerability were identified. The Mixed Methods Appraisal Tool was used to assess study quality. Study content was analysed to identify how structural vulnerability was defined and operationalised.

**Results:**

Thirty‐seven predominantly high‐quality studies published between 2011 and 2024 met inclusion criteria. Structural vulnerability was consistently defined through two interrelated dimensions: as a social positionality (characterised by constrained resilience, limited agency and imposed risks rooted in systemic discrimination and social hierarchies) and as a critical analytic framework for examining structural determinants of health. Quantitative studies predominantly used individual‐level indicators (e.g., income, housing) and cross‐sectional designs. Qualitative studies focused on experiences of structural vulnerability in relation to health outcomes and infrequently translated findings into structural interventions. The most frequently studied outcomes were infectious disease, substance use and mental health.

**Conclusion:**

Structural vulnerability, as a conceptual and empirical lens, reveals how systems produce—and can potentially reduce—health risks. Findings underscore the need for geographically diverse and longitudinal studies, as well as multidimensional measures. Advancing health equity demands critiquing systemic causes of inequities and pursuing justice‐oriented interventions.

**Implications for the Profession:**

Nursing, positioned at the intersection of public health, social sciences and policy, is uniquely equipped to engage structural vulnerability as a critical analytic tool to address health inequities, design interventions and advocate for policy reform.

**Impact:**

*What problem did the study address?* This study addressed a lack of clarity in the definition and operationalization of structural vulnerability in health research.

*What were the main findings?* The definition of structural vulnerability is consistent across quantitative and qualitative studies, but there are marked variations in its operationalization. Quantitative studies predominantly rely on individual‐level indicators, while qualitative studies use it as a theoretical framework to guide analysis, interpret findings and examine structural determinants of health.

*Where and on whom will the research have an impact?* This review offers a clear framing for integrating structural vulnerability in health research in efforts to advance health equity.

**Reporting Method:**

PRISMA (Preferred Reporting Items for Systematic Reviews and Meta‐Analyses) reporting guideline.

**No Patient or Public Involvement:**

This study did not include patient or public involvement in its design, conduct or reporting.

## Abstract (plain language summary)

**Summary**

- What does this paper contribute to the wider global clinical community?
  ○ Clarifies an actionable definition of structural vulnerability for empirical research.
  ○ Encourages structural thinking when considering health outcomes.
  ○ Informs the development of structural competency and interventions that address systems‐level change.
  ○ Provides clinicians, researchers and policymakers with a conceptual foundation to identify, analyse and address entrenched health inequities.

## 1 Introduction

The most pressing threats to health equity are not rooted in biology, but in the structures that shape our lives. Broadly defined, the structural determinants of health include the formal and informal systems that shape how resources are distributed across societies (Heller et al. 2024; Sharif et al. 2022). Structural determinants are influenced by historical, political and cultural forces and are maintained through decisions made by those in positions of power (Heller et al. 2024). As the upstream factors determining the contexts in which people live, work and age, structural determinants are critical to understanding the etiologies of poor health outcomes. With this understanding, health equity scholarship continues to evolve approaches to understanding and addressing the root causes of health inequities (Bailey et al. 2017; Sharif et al. 2022).

New and interdisciplinary concepts have emerged that deepen our understanding of the systemic nature of health inequities. The concept of *structural vulnerability*, introduced in medical anthropology to capture how social hierarchies shape health risks (Quesada et al. 2011), has become increasingly relevant as scholarship on structural determinants of health has expanded (Heller et al. 2024). This paper systematically examines the conceptual framing and operationalization of structural vulnerability in empirical health studies. We begin by outlining the key definitions and frameworks that ground this work.

Definitions of health and health equity, emphasising well‐being and justice, have been foundational to public health scholarship and global health equity discourse. The World Health Organization (WHO) defines health as more than the absence of disease, and health equity as ‘the absence of unfair, avoidable or remediable differences among groups of people’ (Constitution of the World Health Organization 1946; World Health Organization 2025). Building on this foundation, Braveman et al. (2017), in collaboration with the Robert Wood Johnson Foundation, define health equity in terms of the measurable goal to ‘reduce and ultimately eliminate disparities in health and its determinants that adversely affect excluded or marginalised groups’. Together, these definitions advance an understanding of health equity as both a global public health goal and a moral imperative, requiring social and structural transformation to be fully realised.

The WHO's 2010 *Conceptual Framework for Action on the Social Determinants of Health*, commonly referred to as the WHO Social Determinants of Health Framework (WHO SDOH), offers a widely recognised model for the determinants of health inequities (World Health Organization 2010). This framework positions *structural* determinants—macroeconomic and social policies, cultural and societal values, social class and social identities—as antecedents to *intermediary* determinants, which include living conditions, healthcare access, individual behaviours and biological factors (World Health Organization 2010). By presenting an upstream‐to‐downstream model of causality, the WHO SDOH framework serves as a critical starting point for understanding structural determinants as key drivers of well‐being and health.

As efforts to advance health equity and justice expand, the WHO SDOH framework provides a compelling foundation for structural inquiry. Yet despite broad recognition of the role of social, economic and political forces in the perpetuation of health inequities, much empirical health research continues to prioritise intermediary determinants or individual‐level factors over the structural contexts and policies that shape them (Heller et al. 2024). While this focus has yielded valuable data and insights into the patterns of health outcomes, the downstream nature of intermediary determinants often becomes obscured in broad framings of the ‘social determinants of health’ limiting how the salience of structural determinants is understood (Frank et al. 2020; Heller et al. 2024).

Although the persistent emphasis on individual‐level factors is not new, it warrants a renewed critique. When we focus research on determinants of health, identifying *who* is affected and intermediary factors, without adequate attention to *how* or *why* the conditions underlying those outcomes persist, we risk missing an opportunity to understand the structural, or root causes (Frank et al. 2020; Heller et al. 2024). Moving beyond this limitation calls for a conceptual bridge to articulate the dynamic relationship between structural and intermediary determinants. It is along these pathways that the concept of structural vulnerability can guide research, illuminating how systems of power and social inequality shape health.

**FIGURE 1:**
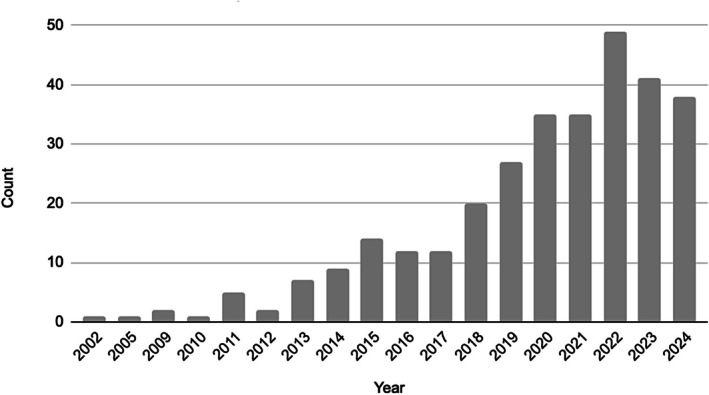
Structural vulnerability citations by year in PubMed.

The term structural vulnerability draws on the linguistic roots of ‘structure’ and ‘vulnerable’ signifying social arrangements that produce susceptibility to harm or injury (Oxford University Press 2023). The term was originally proposed by Quesada et al. (2011) in ethnographic research exploring the health of Latino migrant farm labourers, where it was described as ‘a positionality that imposes physical and emotional suffering on specific population groups in patterned ways’. Since its introduction, the concept has appeared with increasing frequency in health literature (Figure 1), suggesting its application across diverse research questions and settings.

This mixed‐studies review traces the conceptual origins and early applications of structural vulnerability, then synthesises definitions and operational approaches to its use in health research. Our aim is to clarify and distinguish the concept to advance a shared understanding of its analytical utility, and to support its thoughtful application in diverse international and cross‐cultural health contexts. Rather than asserting a conceptual hierarchy, we underscore structural vulnerability's unique contribution to health equity research and address the potential for conceptual flattening that can occur as critical terms gain wider usage.

### 1.1 Structural Vulnerability: Conceptual Origins and Early Applications

Structural vulnerability builds upon the concept of ‘*structural violence*’, introduced in the 1960s by peace studies scholar Johan Galtung. Structural violence was defined as a form of social injustice manifesting from disparities in power and material deprivation (Galtung 1969). Building from this definition, Paul Farmer, physician and medical anthropologist, stands out as a scholar who brought the concept of structural violence to increasing popularity in global health discourse. Farmer was instrumental in reframing health as a human right; he argued that depriving people of access to basic needs constitutes a form of violence (Farmer 2004).

Despite Farmer's influential use of the concept, structural violence has seen limited uptake in health care and research, in part because it has been viewed as too abstract or inflammatory (Quesada et al. 2011). Recognising both its conceptual contributions and rhetorical limits, and building upon evolving theorizations of vulnerability, Quesada et al. (2011) introduced structural vulnerability. In their conception, structural vulnerability arises from the interaction between personal traits (e.g., appearance, behaviour or cognitive status) with cultural norms and power differentials that organise a hierarchical society.

The choice of terminology is significant. Structural vulnerability aligns with broader critiques of population health scholarship that caution the use of terms like ‘vulnerable’, ‘vulnerability’ and ‘vulnerable populations’. These terms risk implying inherent weakness, passivity or deficiency, obscuring the structural conditions that produce inequities (Garrett and Altman 2024; Haalboom and Natcher 2012). Instead, structural vulnerability, as presented in the work by Quesada et al. (2011), forefronts external, systemic forces that constrain agency and reproduce hardship, especially in societies like the United States (U.S.), where social hierarchies and free market logics intensify privilege and disadvantage (Quesada et al. 2011; Holmes 2011). In this framing, the cause of vulnerability is considered to originate outside the individual, and the emphasis on structures is inherent in the term itself.

When structural vulnerability was introduced for use in clinical contexts, it was not intended as a descriptive or rhetorical label for characterising risk, nor as a diagnostic category. Rather, it was proposed as a conceptual tool useful to uncover a wider range of causes of health circumstances and outcomes, and for targeting resources (Quesada et al. 2011). Building on this understanding and bridging theory and clinical practice, Bourgois et al. (2017) operationalised the concept through the Structural Vulnerability Assessment Tool (SVAT), a set of qualitative probes designed to help clinicians identify and address structural barriers to health across eight key domains (see Bourgois et al. 2017). Through case studies, Bourgois et al. (2017) demonstrated how the SVAT supported clinicians and multidisciplinary teams to reframe individual‐level challenges in relation to their structural determinants. For example, in treating a 22‐year‐old African American male with paraplegia in the aftermath of interpersonal violence, the SVAT guided care beyond the clinical encounter to include advocacy for accessible housing, legal protections and transportation support. These interventions directly addressed the structural barriers shaping his healing, recovery and reintegration into his community (Bourgois et al. 2017).

Originally rooted in ethnographic research (Holmes 2011; Quesada et al. 2011) and later operationalised for clinical use (Bourgois et al. 2017), early scholarship on structural vulnerability underscores its theoretical and ethical value. It reframes health inequities as consequences of entrenched social inequality and systemic injustice. This review builds on that foundation by synthesising how structural vulnerability has been applied in empirical health research and by evaluating its contributions, and potential, for advancing health equity‐oriented scholarship.

## 2 Literature Review

### 2.1 Aims

This review was conducted with two primary aims: (1) to synthesise how structural vulnerability has been defined and operationalised in empirical health studies; and (2) to identify variations and inconsistencies in these definitions and applications. Based on these insights, we define key dimensions of structural vulnerability, clarify its conceptual and methodological contributions, and outline implications for its future use in health research.

## 3 Methods

### 3.1 Design

This mixed‐studies systematic review was conducted using a parallel‐results convergent synthesis design (Hong et al. 2017), in which quantitative and qualitative studies are evaluated independently, with integrated findings presented in the discussion. It follows the Preferred Reporting Items for Systematic Reviews and Meta‐Analyses (PRISMA) 2020 statement and checklist to meet established criteria for reporting systematic reviews in a transparent, reproducible manner (Page et al. 2021).

### 3.2 Search Methods

In consultation with a research librarian relevant literature was identified through a search of PubMed, Embase, Scopus and CINAHL and the terms ‘structural* and/or vulnerab*’ The search had no lower date limit and included all relevant studies through the end of 2024. A detailed overview of the search strategy is provided in Table S1.

### 3.3 Inclusion and Exclusion Criteria

Peer‐reviewed studies published in English and conducted in the U.S. were included. Geographic limits focused on the U.S. due to its unique sociopolitical history, systemic inequalities and healthcare system (Bailey et al. 2017; Sharif et al. 2022). Studies were included if they either (1) operationalised structural vulnerability as a measurable construct, or (2) explicitly noted the use of structural vulnerability as a framework or conceptual lens to investigate a health outcome or health‐related factor. Abstracts, grey literature, case studies, non‐human studies and research not examining or measuring structural vulnerability in health contexts were excluded.

### 3.4 Search Outcome

**FIGURE 2:**
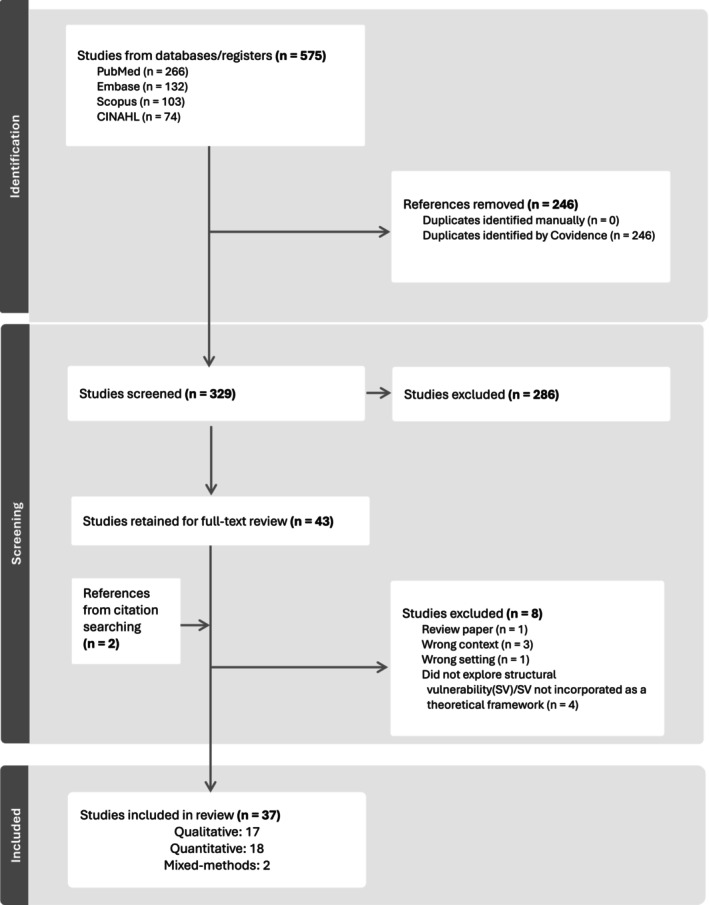
Literature search flow diagram.

The search yielded 266 citations from PubMed, 132 from Embase, 103 from Scopus and 74 from CINAHL. A literature search flow diagram is included in Figure 2. All citations (*n* = 575) were uploaded into Covidence, a web‐based software platform that streamlines systematic and other literature review processes (Covidence 2024). After the removal of duplicate citations (*n* = 246), the remaining 329 citations were screened by title and abstract by the first author. Forty‐three articles were identified for full‐text review, along with two additional articles identified through citation searching, for a total of forty‐five. Of these, thirty‐six met the inclusion criteria. A second reviewer (SJ) conducted a blinded verification of inclusions and exclusions against the predetermined criteria, identifying one additional article. After consensus, thirty‐seven articles were retained for this mixed‐studies review.

### 3.5 Quality Appraisal

The Mixed Methods Appraisal Tool (MMAT) was used for quality appraisal. All studies were evaluated according to two standard screening questions, followed by five appraisal questions specific to their respective research methodologies and study designs. The current version of the MMAT recommends a narrative appraisal rather than a numerical score (Hong et al. 2018).

### 3.6 Data Abstraction and Synthesis

A comprehensive review of all studies was conducted prior to data extraction and synthesis. Table 1 presents an overview of the included studies. A detailed account of each study, including characteristics and key findings, is presented in Table S2.

**TABLE 1:** Studies applying the concept of structural vulnerability in empirical health studies.

Citation	Study purpose	Sample	Setting	Study type and design
Beckham et al. (2022)^a^ Latent classes of polysubstance use and associations with HIV risk and structural vulnerabilities among cisgender women who engage in street‐based transactional sex in Baltimore city	To describe patterns of substance use and associations with HIV‐related risks and structural vulnerabilities among cisgender women engaged in street‐based sex work	244 cisgender women involved in street‐based sex work	Baltimore, MD	Quantitative Cross‐sectional
Brantley, Footer, et al. (2017) Identifying patterns of social and economic hardship among structurally vulnerable women: a latent class analysis of HIV/STI Risk	To profile experiences of structural vulnerability by identifying distinct patterns of co‐occurring social and economic disadvantage; and to investigate how different indicators of structural vulnerability cluster together and are associated with drug use and sexual risk behaviour	117 women working in an exotic dance club	Baltimore, MD	Quantitative Cross‐sectional
Footer et al. (2020)^a^ Entry to sex trade and long‐term vulnerabilities of female sex workers who enter the sex trade before the age of eighteen	To analyse how structural factors, such as food insecurity, and homelessness manifest in the present day lives of those who traded sex under 18 years of age versus later in life, in addition to exploring the relationship of homelessness and food insecurity for cisgender women involved in sex work and current HIV risk behaviours	250 cisgender women involved in street based sex work	Baltimore, MD	Quantitative Cross‐sectional
Glick et al. (2020)^a^ Structural vulnerabilities and HIV risk among sexual minority female sex workers (SM‐FSW) by identity and behaviour in Baltimore, MD	To examine the differences in structural vulnerabilities and HIV risk drivers between sexual minority women, by identity and behaviour, and their heterosexual counterparts among cisgender women involved in sex work	247 cisgender women involved in street based sex work	Baltimore, MD	Quantitative Cross‐sectional
James and Horne (2024) Examining barriers to dental, medical, mental and vision healthcare access, attitudes towards seeking healthcare, and internalised racism among Black Americans	To examine if internalised racism explained the relationship between healthcare access barriers and health attitudes	780 Black American adults	National	Quantitative Cross‐sectional
Jegede et al. (2021) Perceived barriers to access care, anticipated discrimination and structural vulnerability among African Americans with substance use disorders	To assess perceived structural vulnerability, perceived barriers to access care and anticipated discrimination among African American patients currently in inpatient treatment for substance use disorder (SUD)	58 African American patients receiving inpatient treatment for a SUD	Brooklyn, NY	Quantitative Cross‐sectional
King, Gamarel, et al. (2023) Structural needs, substance use, and mental health among transgender and non‐binary young adults in the San Francisco Bay Area: Findings from the Phoenix study	To describe sociodemographic patterns in self‐reported structural needs and unmet structural needs, and to examine the relationship between structural needs, mental health, and substance use outcomes among transgender and non‐binary young adults	215 transgender and non‐binary young adults	San Francisco, CA	Quantitative Cross‐sectional
King, Jadwin‐Cakmak, et al. (2023) Structural vulnerability as a conceptual framework for transgender health research: findings from a community needs assessment of transgender women of colour in Detroit	To demonstrate the utility of using a structural vulnerability framework to understand distributions of adverse health outcomes among transgender populations; and to explore the relationship between structural vulnerability and mental health and substance use outcomes	60 transgender women of colour	Detroit, MI	Quantitative Cross‐sectional
Lim et al. (2019)^a^ Severe food insecurity, gender‐based violence, homelessness and HIV risk among street‐based female sex workers in Baltimore, Maryland	To identify correlates—structural vulnerability and health factors—of severe food insecurity among street‐based FSW in Baltimore, Maryland	249 cisgender women involved in street based sex work	Baltimore, MD	Quantitative Cross‐sectional
Organista et al. (2019) Working and living conditions and psychological distress in Latino migrant day labourers	To test a model of hypothesised pathways between working and living conditions and multiple forms of psychological distress	344 Latino migrant day labourers	San Francisco and Berkeley, CA	Quantitative Cross‐sectional
Pérez‐Figueroa et al. (2022) Housing instability, structural vulnerability and non‐fatal opioid overdoses among people who use heroin in Washington Heights, New York City	To evaluate the association between key contextual factors and experiencing a non‐fatal opioid overdose among people who use heroin	101 people who use heroin	Washington Heights, New York	Quantitative Cross‐sectional
Reilly et al. (2015) Structural vulnerabilities to HIV/STI risk among female exotic dancers in Baltimore, Maryland	To characterise indicators of structural vulnerability associated with HIV/STI risk behaviour and explore the effect of accumulated vulnerability on the likelihood of dancers' engagement in risky behaviour	101 female exotic dancers	Baltimore, MD	Quantitative Cross‐sectional
Schneider et al. (2024) Sleep‐related impairment among people who use opioids: The critical role of structural vulnerability	To examine differences in sleep context and characteristics and to assess associations of sociodemographic and substance use characteristics with sleep related impairments	170 people who use opioids	Anne Arundel County, MD	Quantitative Cross‐sectional
Schneider et al. (2022)^b^ Understanding the longitudinal relationship between substance use and violent victimisation among street‐based women who exchange sex in Baltimore, Maryland	To explore the longitudinal relationship between violence and drug use among a sample of women who exchange sex from Baltimore City, Maryland; and to examine how baseline indicators of socioeconomic and structural vulnerability are associated with drug use and experiences of violence	251 cisgender women engaged in street based transactional sex	Baltimore, MD	Quantitative Longitudinal
Sherman et al. (2024) Stigma, social and structural vulnerability, and mental health among transgender women: A partial least square path modelling analysis	To examine the relationships between co‐occurring stigma exposure, social and structural vulnerabilities, and mental health and identify common characteristics among transgender women to improve intervention tailoring for this group	1418 transgender women.	Various US regions Midwest, Northeast, South, West	Quantitative Cross‐sectional
Sherman et al. (2019)^a^ Drivers of HIV infection among cisgender and transgender female sex worker populations in Baltimore city: results from the SAPPHIRE Study	To better characterise the shared and distinct structural vulnerabilities of transgender female sex workers and cisgender female sex workers; and unpack how socio‐structural factors synergistically and independently drive women's entry into sex work and shape the context in which HIV infection and risk‐taking occur	313 cisgender and transgender women involved in street based sex work	Baltimore, MD	Quantitative Cross‐sectional
Tomko et al. (2023)^b^ Mental health and HIV risk differs by co‐occurring structural vulnerabilities among women who sell sex	To study the relationship between co‐occurring structural vulnerabilities and mental health in a population of female sex workers at high risk for HIV using latent class analysis	385 cisgender women involved in street based transactional sex	Baltimore, MD	Quantitative Cross‐sectional
Urquhart et al. (2021)^a^ Associations of cumulative violence and structural vulnerability with restless sleep among female sex workers in Baltimore, Maryland	To characterise associations of individual, interpersonal and structural factors with frequent restless sleep among female sex workers	236 cisgender women involved in street based sex work	Baltimore, MD	Quantitative Cross‐sectional
Friedman et al. (2019) Structural vulnerability to narcotics‐driven firearm violence: An ethnographic and epidemiological study of Philadelphia's Puerto Rican inner city	To characterise the structural vulnerability of the Puerto Rican community in Philadelphia to gun violence using a mixed methods approach, and to present an assessment of firearm violence in these communities that is quantitatively rigorous while conveying the lived experience of the human suffering reflected in the macro statistics	Sample included an unspecified number/‘dozens of respondents’	Philadelphia, PA	Mixed Methods Exploratory sequential
Friedman et al. (2021) Intersectional structural vulnerability to abusive policing among people who inject drugs: A mixed methods assessment in California's Central Valley	To assess how experiences with abusive or violent policing among people who inject drugs relate to intersectional structural vulnerability	494 people who inject drugs	Fresno and Kern Counties, California	Mixed Methods Exploratory sequential
Arnold et al. (2021) Structural vulnerability and occupational injury among Latinx child farmworkers in North Carolina	To describe the broad experience of personal and observed workplace injuries of Latinx child farmworkers in North Carolina. To highlight child labour in one of the most hazardous industries	30 child farmworkers	North Carolina	Qualitative Semi‐structured interviews
Arnold et al. (2024) Essential(ly forgotten) workers: Latine youth farmworkers during the COVID‐19 pandemic	To describe and contextualise Latine youth farmworkers experiences of work safety during the first 2 years of the COVID‐19 pandemic by drawing from interviews with education, health and non‐profit advocacy service providers and Latine youth farmworkers	24 Latine youth farmworkers	North Carolina	Qualitative Semi‐structured interviews
Brantley, Kerrigan, et al. (2017) Experiences of structural vulnerability among exotic dancers in Baltimore, Maryland: Co‐occurring social and economic antecedents of HIV/STI risk	To uncover how structural vulnerability is experienced at the individual level and to examine the interplay of structural drivers before and after initial entry into the exotic dance club environment	24 exotic dancers working in Baltimore City and County exotic dance clubs	Baltimore City and County, Maryland	Qualitative Longitudinal Semi‐structured interviews
Chang et al. (2019) Narratives of people who inject drugs on factors contributing to opioid overdose	To examine the narratives of people who inject drugs surrounding their recent overdose experiences	40 people who inject drugs	San Francisco, California	Qualitative Semi‐structured interviews
Collins et al. (2024) ‘Everywhere I call, there's nothing available’: Understanding the alcohol treatment landscape and needs among unstably housed people who use alcohol in Rhode Island	To understand the alcohol‐related treatment and support needs of unstably housed individuals with high‐intensity alcohol use in Rhode Island	25 unstably housed individuals who self‐reported heavy alcohol use	Rhode Island	Qualitative Semi‐structured interviews/participant observation
Haas et al. (2018) Bringing a structural perspective to work: Framing occupational safety and health disparities for nursing assistants with work‐related musculoskeletal disorders	Utilise a structural vulnerability framework to more broadly define the hazards influencing high work‐related musculoskeletal disorders rates among Certified Nursing Assistants (CNAs); and to demonstrate the utility of structural vulnerability theory in occupational safety and health (OSH) research more broadly	26 CNAs working in nursing and residential care facilities	Washington state	Qualitative Semi‐structured interviews
Holmes (2011) Structural vulnerability and hierarchies of ethnicity and citizenship on the farm	To analyse hierarchies of ethnicity and citizenship, structural vulnerability and health disparities in agriculture in the United States	Unspecified number of participants	Skagit County, Washington	Qualitative Ethnography
Kohut et al. (2024) A qualitative study of how structural vulnerability shaped COVID‐19 testing behaviours in Portland, Maine	This study sought to understand and reduce the barriers to COVID‐19 testing faced by structurally vulnerable populations in Portland, Maine, with a focus on individuals experiencing homelessness, immigrants, and those who are low‐income or uninsured	34 members of structurally vulnerable populations	Portland, Maine	Qualitative Semi‐structured interviews
Manser et al. (2024) Homelessness and type 2 diabetes: A qualitative study of facilitators and barriers to self‐management and medication adherence	To explore barriers and facilitators to diabetes medication adherence and self‐management among people with type 2 diabetes who have experienced homelessness	26 participants with type 2 diabetes and lived experience of homelessness	Minneapolis, MN	Qualitative Focus groups/semi‐structured interviews
McKenna (2014) Navigating the risk environment: Structural vulnerability, sex and reciprocity among women who use methamphetamine	To explore how women who use methamphetamine navigate sexual relationships, survival strategies and drug acquisition in the context of structural vulnerability and risk environments, particularly focusing on exchange sex and reciprocity	Over 30 women who use methamphetamine	Denver, Colorado	Qualitative Ethnography
Organista et al. (2013) Sexual health of Latino migrant day labourers under conditions of structural vulnerability	To explore the sexual health and well‐being of Latino migrant day labourers with attention to conditions of structural vulnerability	51 Latino migrant day labourers	San Francisco and Berkeley, CA	Qualitative Ethnography
Tulimiero et al. (2021) Overcoming barriers to health care access in rural Latino communities: An innovative model in the Eastern Coachella Valley	To explore community health priorities and barriers to Latino immigrants' health care services use in rural communities, and participants' ideal model for health care services delivery	Unspecified number of Latino immigrants	Eastern Coachella Valley California	Qualitative Focus groups/Semi‐structured interviews
Westbrook (2024) The embodiment of exclusionary displacement pressure: Intersections of housing insecurity and mental health in a Hispanic/Latinx immigrant neighbourhood	To examine how exclusionary displacement pressure shapes low‐income residents' health and wellbeing over time	35 residents in a predominantly low‐income Hispanic/Latinx immigrant neighbourhood	Denver, Colorado	Qualitative Ethnography
Worby et al. (2014) Structural vulnerability and problem drinking among Latino migrant day labourers in the San Francisco Bay Area	To explore how Latino migrant day labourers distinguish problem drinking from other kinds of drinking, and what actions they take in response to problem drinking	51 Latino migrant day labourers	San Francisco and Berkeley, CA	Qualitative Ethnography
Yang et al. (2014) ‘What matters most’: A cultural mechanism moderating structural vulnerability and moral experience of mental illness stigma	To understand Chinese immigrants' experiences with mental illness stigma and mental health disparities and to identify how the interaction between structural discrimination and cultural engagements might shape stigma	50 Chinese immigrants	New York City, NY	Qualitative Semi‐structured interviews
Young et al. (2022) The structural impacts of enforcement policy on Latino immigrant health	To identify the structural factors that may be mechanisms between enforcement policy and immigrant health through the perspective of Latino immigrants	14 Latino immigrants	Two southern California counties	Qualitative Unstructured interviews
Zhen‐Duan et al. (2022) Using a structural vulnerability framework to understand the impact of COVID‐19 on the lives of Medicaid beneficiaries receiving substance use treatment in New York City	To understand how the COVID‐19 pandemic impacted low‐income individuals with SUD and how people adjusted to SUD treatment changes during ‘stay‐at‐home’ orders in NYC	20 adult participants, enrolled in Medicaid, receiving outpatient treatment for substance use	New York City, NY	Qualitative Semi‐structured interviews

^a^ Denotes secondary data analysis of the SAPPHIRE study: Silberzahn et al. (2020). Barriers and facilitators to retaining a cohort of street‐based cisgender female sex workers recruited in Baltimore, Maryland, USA: results from the SAPPHIRE study.

^b^ Denotes secondary data analysis of the EMERALD study: Silberzahn et al. (2021) The EMERALD (Enabling Mobilisation, Empowerment, Risk Reduction and Lasting Dignity) Study: Protocol for the design, implementation, and evaluation of a community‐based combination HIV prevention intervention for female sex workers in Baltimore, Maryland.

Data synthesis focused on how structural vulnerability has been defined and operationalised. Definitions were thematically analysed to identify recurring concepts and emergent themes, moving beyond simple summarisation to examine how structural vulnerability has been conceptualised across empirical studies (Braun and Clarke 2022). Approaches to operationalization were synthesised narratively to capture how the concept was measured or applied within different study designs (Pluye and Hong 2014). In quantitative studies, indicators used to measure structural vulnerability were categorised and grouped by frequency of use.

## 4 Results

The thirty‐seven studies in this review include quantitative (*n* = 18), qualitative (*n* = 17) and mixed methods (*n* = 2) research. Twenty‐seven articles were appraised as high quality, and ten as moderate to high quality (Table 2).

**TABLE 2:** Quality appraisal—Mixed Methods Appraisal Tool (MMAT), version 2018.

Quantitative descriptive studies							
Citation	Are there clear research questions?	Do the collected data allow to address the research questions?	Is the sampling strategy relevant to address the research question?	Is the sample representative of the target population?	Are the measurements appropriate?	Is the risk of non‐response bias low?	Is the statistical analysis appropriate to answer the research question?
Jegede et al. (2021)	Y	Y	Limited sample size	Y	Y	Y	Y

Quantitative non‐randomised studies							
Citation	Are there clear research questions?	Do the collected data allow to address the research questions?	Are the participants representative of the target population?	Are the measurements appropriate regarding both the outcome and intervention (exposure)?	Are there complete outcome data?	Are the confounders accounted for in the design and analysis?	During the analysis is the intervention administered (or exposure occurred) as intended?
Beckham et al. (2022)	Y	Y	Y	Y	Y	Y	Y
Brantley, Kerrigan, et al. (2017)	Y	Y	Y	Y	Y	Y	Y
Footer et al. (2020)	Y	Y	Y	Y	Y	Y	Y
Glick et al. (2020)	Y	Y	Y	Indicators of HIV risk drivers are unclear. Validity and reliability of depression and PTSD not measures are not reported	Y	Y	Y
James and Horne (2024)	Y	Y	Y	Some measures lacked formal validation; some demonstrated low internal consistency	Y	Y	Y
King, Gamarel, et al. (2023)	Y	Y	Y	Y	Y	Y	Y
King, Jadwin‐Cakmak, et al. (2023)	Y	Y	Small sample recruited during the COVID‐19 pandemic; limited the generalizability	Indicators of structural vulnerability are limited given the target population	Y	Y	Y
Lim et al. (2019)	Y	Y	Y	Unclear measure of food insecurity; validity/reliability of measures not reported	Y	Y	Y
Organista et al. (2019)	Y	Y	Y	Y	Y	Y	Y
Pérez‐Figueroa et al. (2022)	Y	Y	Y	Structural vulnerability indicators are not clearly delineated	Y	Y	Y
Reilly et al. (2015)	Y	Y	Y	Y	Y	Y	Y
Schneider et al. (2022)	Y	Y	Y	Y	Y	Y	Y
Schneider et al. (2024)	Y	Y	Y	Y	Y	Y	Y
Sherman et al. (2019)	Y	Y	Y	Y	Y	Y	Y
Sherman et al. (2024)	Y	Y	Y	Internal consistency of the measures for mental health outcomes not reported	Missing data proportions (8.8%–17%) varied by group	Y	Y
Tomko et al. (2023)	Y	Y	Y	Y	Y	Y	Y
Urquhart et al. (2021)	Y	Y	Y	Number of traumas experienced reported as statistically significant finding; measure is not defined or described	Y	Y	Y

Mixed methods studies							
Citation	Are there clear research questions?	Do the collected data allow to address the research questions?	Is there an adequate rationale for using a mixed methods designed to address the research question?	Are the different components of the study effectively integrated to answer the research question?	Are the outputs of the integration of qualitative and quantitative components adequately interpreted?	Are divergences and inconsistencies between quantitative and qualitative results adequately addressed?	Do the different components of the study adhere to the quality criteria of each tradition in the methods involved?
Friedman et al. (2019)	Y	Y	Y	Y	Y	Y	Y
Friedman et al. (2021)	Y	Y	Y	Y	Y	Y	Y

Qualitative studies							
Citation	Are there clear research questions?	Do the collected data allow to address the research questions?	Is the qualitative approach appropriate to answer the research question?	Are the qualitative data collection methods adequate to address the research question?	Are the findings adequately derived from the data?	Is the interpretation of results sufficiently substantiated by data?	Is there coherence between qualitative data sources, collection, analysis and interpretation?
Arnold et al. (2021)	Y	Y	Y	Y	Y	Y	Y
Arnold et al. (2024)	Y	Y	Y	Y	Y	Y	Y
Brantley, Footer, et al. (2017)	Y	Y	Y	Y	Y	Y	Y
Chang et al. (2019)	Y	Y	Y	Y	Y	Y	Y
Collins et al. (2024)	Y	Y	Y	Y	Y	Y	Y
Haas et al. (2018)	Y	Y	Y	Y	Y	Y	Y
Holmes (2011)	Y	Y	Y	Y	Y	Y	Y
Kohut et al. (2024)	Y	Y	Y	Y	Y	Y	Y
Manser et al. (2024)	Y	Y	Y	Y	Y	Y	Y
McKenna (2014)	Y	Y	Y	Y	Y	Y	Y
Organista et al. (2013)	Y	Y	Y	Y	Grounded theory approach: theory generated not made clear	Y	Y
Tulimiero et al. (2021)	Y	Y	Y	Y	Y	Y	Y
Westbrook (2024)	Y	Y	Y	Y	Y	Y	Y
Worby et al. (2014)	Y	Y	Y	Y	Y	Y	Y
Yang et al. (2014)	Y	Y	Y	Y	Y	Y	Y
Young et al. (2022)	Y	Y	Unstructured open ended interviews did not lead to data about health status	Y	Y	Y	Y
Zhen‐Duan et al. (2022)	Y	Y	Y	Y	Y	Y	Y

### 4.1 Study Characteristics

#### 4.1.1 Populations of Interest

The populations of interest varied (Table S2). The studies most frequently focused on people who engaged in sex work (*n* = 8), followed by individuals who used illicit substances or had a substance use disorder (*n* = 7). Other populations included: immigrants to the U.S. (*n* = 3: Latino, *n* = 2; Chinese, *n* = 1), migrant labourers (*n* = 3), and agricultural workers (*n* = 3), transgender or gender non‐conforming individuals (*n* = 3), residents of low‐income areas (*n* = 3), exotic dancers (*n* = 3), certified nursing assistants (*n* = 1), Black American adults (*n* = 1) and people experiencing homelessness (*n* = 1). One study included a heterogeneous sample of structurally vulnerable participants. It is important to note that some overlap exists among these categories; for example, participants who engaged in sex work may also have used illicit substances or had a substance use disorder (see Table S2).

#### 4.1.2 Health Outcomes or Related Factors

Structural vulnerability was studied or measured in relation to specific health outcomes or related factors. The most common were HIV/STI risk (*n* = 10), mental health and well‐being (*n* = 7), and substance use (*n* = 5). Other health factors included occupational injury (*n* = 3), exposure to violence (*n* = 3: interpersonal *n* = 2; police violence *n* = 1), access to healthcare (*n* = 3), the COVID‐19 pandemic (*n* = 2), sleep quality (*n* = 2), type 2 diabetes (*n* = 1) and food insecurity (*n* = 1).

#### 4.1.3 Study Designs

Among the eighteen quantitative studies, most were cross‐sectional (*n* = 16), and many utilised secondary data analyses (*n* = 13). A majority of the seventeen qualitative studies involved one‐time interviews or focus groups (*n* = 11); one study was longitudinal (conducted over 6 months), and five were ethnographies. The two mixed methods studies used exploratory sequential designs. Across methodological approaches, several (*n* = 16) were co‐designed with community advisory boards or incorporated a community‐based participatory research (CBPR) approach (Table S2).

### 4.2 Quantitative Synthesis

#### 4.2.1 Defining Structural Vulnerability in Quantitative Health Research

Quantitative researchers defined structural vulnerability through four key themes: (1) social positionality, (2) constrained resilience and limited agency, (3) imposed risk and (4) an analytic framework. These themes inherently overlap, underscoring the concept's complexity and nuance.

##### 4.2.1.1 Social Positionality

Social positionality is defined as ‘the occupation or adoption of a particular position in relation to others, usually with reference to issues of culture, ethnicity or gender’ (Oxford University Press 2025a). Studies structural vulnerability in this way emphasised how social positioning—particularly undocumented status and socioeconomic marginalisation—shaped access to resources and health outcomes (Organista et al. 2019; Pérez‐Figueroa et al. 2022; Sherman et al. 2019; Urquhart et al. 2021).

##### 4.2.1.2 Constrained Resilience and Limited Agency

Resilience refers to the capacity to recover from adversity, while agency, or the capacity to act, is intertwined with autonomy and self‐determination (Hitlin and Long 2009; Southwick et al. 2014). Some authors define structural vulnerability as constrained resilience and limited agency, describing how institutional and systemic forces diminish individuals' capacity by reinforcing exclusionary practices and imposing stigmatising social norms. These processes, in turn, limit an individual's ability to act, make decisions, respond to harm or advocate for their well‐being and ultimately exert control over their lives (Glick et al. 2020; Sherman et al. 2024; Tomko et al. 2023).

##### 4.2.1.3 Imposed Risk

A defining characteristic of structural vulnerability is the idea of risk, defined as exposure to ‘the possibility of loss, injury, or other adverse or unwelcome circumstance’ (Oxford University Press 2025b, 2025c). Several studies defined structural vulnerability as the imposition of risks that negatively impact health. Jegede et al. (2021), for example, framed structural vulnerability as the risks imposed upon African American patients in substance use treatment as they encountered barriers to care and anticipated racial discrimination. Additionally, risk emerged in definitions emphasising the cumulative impact of multiple social and economic disadvantages (e.g., poverty and legal precarity) (Brantley, Kerrigan, et al. 2017; Reilly et al. 2015).

##### 4.2.1.4 A Framework for Analysis

Several studies define structural vulnerability as an analytic framework for examining how cultural, economic and systemic forces shape health risks (Friedman et al. 2021; Footer et al. 2020; James and Horne 2024; King, Gamarel, et al. 2023; King, Jadwin‐Cakmak, et al. 2023). Researchers identified structural vulnerability for their analysis of social inequality, economic exploitation and stigma—particularly those rooted in cisgenderism and ethno‐racism (King, Gamarel, et al. 2023; King, Jadwin‐Cakmak, et al. 2023). This conceptual framing also informed methodological decisions, including the selection of study variables and data analysis.

#### 4.2.2 Operationalising Structural Vulnerability in Quantitative Health Research

The quantitative studies operationalised structural vulnerability in multiple ways, most commonly as a measurable construct and less frequently as an analytic framework. To measure structural vulnerability, researchers identified indicators similar to metrics used in measurements of socioeconomic status (e.g., income, education, employment). Two studies utilised the Structural Vulnerability Assessment Tool (SVAT), and one study used a novel survey to assess structural needs. These indicators were then incorporated into statistical analyses to examine their associations with health‐related outcomes (see Table S2).

##### 4.2.2.1 Individual Indicators of Structural Vulnerability

Sixteen quantitative studies and one mixed methods study identified individual‐level indicators to measure structural vulnerability. The following categories of indicators are listed in order of how frequently they appeared across studies: housing and living conditions (*n* = 16), food insecurity (*n* = 12), experiences with the criminal justice system (*n* = 8), educational attainment (*n* = 7), financial status/security (*n* = 7), receipt of social services/support (*n* = 6), employment and working conditions (*n* = 4), health status and health care access (*n* = 4), exposure to violence (*n* = 3) and discrimination (*n* = 2). These indicators were most often used as independent variables in analyses examining associations with health outcomes. Table S3 provides a detailed overview of the indicators and their descriptions (Beckham et al. 2022; Brantley, Kerrigan, et al. 2017; Footer et al. 2020; Friedman et al. 2021; Glick et al. 2020; King, Gamarel, et al. 2023; King, Jadwin‐Cakmak, et al. 2023; Jegede et al. 2021; Lim et al. 2019; Organista et al. 2019; Reilly et al. 2015; Schneider et al. 2024, 2022; Sherman et al. 2024, 2019; Tomko et al. 2023; Urquhart et al. 2021).

##### 4.2.2.2 Adaptations of the Structural Vulnerability Assessment Tool

Two studies adapted the SVAT for quantitative analysis by developing survey instruments aligned with the eight domains developed by Bourgois et al. (2017)—financial security, residence, risk environment, food access, social network, legal status, education and discrimination. In a study of African American inpatients with substance use disorder, Jegede et al. (2021) developed a survey based on the SVAT domains, modifying the discrimination domain to exclude items intended to elicit providers' or researchers' perceptions of their own bias. In a study with transgender women of colour, King, Jadwin‐Cakmak, et al. (2023) identified constructs corresponding to the eight SVAT domains and combined items from validated scales (e.g., the Gender Minority Stress and Resilience Measure) with SVAT questions to quantify associations among structural vulnerability indicators, mental health outcomes and substance use.

##### 4.2.2.3 Structural Needs Scale

Structural vulnerability was measured by metrics of structural needs in a novel scale developed by King, Gamarel, et al. (2023). They developed summative scales to quantify met and unmet needs, using indicators of structural vulnerability (e.g., housing, food assistance, health insurance), to examine how self‐reported needs related to substance use and mental health outcomes. The scales showed acceptable internal reliability, with Cronbach's alpha values of 0.77 (structural needs) and 0.74 (unmet needs). Total scores, ranging from 0 to 9 for each scale, were analysed as continuous variables in regression models.

##### 4.2.2.4 Statistical Modelling of Structural Vulnerability

Four studies used latent class analysis to quantify structural vulnerability and incorporated these classifications into statistical models (Beckham et al. 2022; Brantley, Kerrigan, et al. 2017; King, Jadwin‐Cakmak, et al. 2023; Tomko et al. 2023). King, Jadwin‐Cakmak, et al. (2023) found that relationships between three vulnerability ‘classes’ and health outcomes were more apparent than when analysing individual indicators. Three other studies used structural equation modelling, partial least squares path modelling, or path analysis to examine relationships between indicators of structural vulnerability and health outcomes. These studies identified significant associations with outcomes such as depression, violent victimisation and mental health symptom severity (Organista et al. 2019; Schneider et al. 2022; Sherman et al. 2024).

##### 4.2.2.5 Structural Vulnerability as an Analytical Framework

Two studies used structural vulnerability solely as a framework without direct measurement. One examined internalised racism and healthcare access (James and Horne 2024), while another studied opioid overdose and housing instability (Pérez‐Figueroa et al. 2022). Using structural vulnerability as a framework, these studies highlighted the role of social and systemic factors in the health outcomes of their study sample.

### 4.3 Qualitative Synthesis

#### 4.3.1 Defining Structural Vulnerability in Qualitative Health Research

Definitions of structural vulnerability in qualitative studies generally echo those in quantitative research; however, they offer additional nuance in characterising the concept. Three overarching themes emerged: (1) the impact of structural violence and discrimination, (2) the human‐made patterning of social suffering and (3) structural vulnerability as a lens that shifts the analytical gaze. Together, these themes highlight the relational, historically rooted and action‐oriented nature of how structural vulnerability is defined.

##### 4.3.1.1 The Impact of Structural Violence and Discrimination

Across multiple studies, structural vulnerability is defined as encompassing the lived consequences of systemic disadvantage stemming from structural violence and pervasive discrimination. It emerges in the context of macro‐level forces—such as economic exploitation, institutional racism and housing instability—that are embedded in and perpetuated by discriminatory ideologies and policies (Arnold et al. 2024; Manser et al. 2024; Yang et al. 2014; Westbrook 2024). Structural vulnerability, in turn, undermines opportunities for health by shaping factors such as decision‐making and chronic disease management, ultimately contributing to persistent health inequities (Brantley, Footer, et al. 2017; Haas et al. 2018; Manser et al. 2024).

##### 4.3.1.2 The Human‐Made Patterning of Disadvantage and Suffering

Structural vulnerability is not an inherent condition, but a phenomenon produced and reproduced through human action. The concept was defined as ‘human‐made’ (Chang et al. 2019), and often with an implicit emphasis on the role of individuals, institutions and policy actors in actively shaping conditions of disadvantage (Arnold et al. 2021; Chang et al. 2019; Collins et al. 2024; Friedman et al. 2019; Kohut et al. 2024; McKenna 2014; Organista et al. 2013; Worby et al. 2014). These definitions converge on the recognition that people create the very structures that produce vulnerability through choices about resource allocation, exclusion, and the normalisation of systemic neglect. This framing defines the concept by acknowledging the accountability of the social actors who maintain—and who could challenge—inequitable systems.

##### 4.3.1.3 Shifting the Analytical Gaze

Several studies defined structural vulnerability using action‐oriented phrases such as ‘shifts the focus’, ‘situates’, ‘acknowledges’ or ‘trains the gaze’. These definitions align with those found in quantitative research; however, the language indicates a deliberate reorientation that prompts an epistemological shift—one that compels recognition of the social, political and economic forces shaping health outcomes (Holmes 2011; Tulimiero et al. 2021; Young et al. 2022; Zhen‐Duan et al. 2022).

#### 4.3.2 Operationalising Structural Vulnerability in Qualitative Health Research

In qualitative research, structural vulnerability informed the development of interview guides, guided participant observation and the process of data analysis. Two interconnected themes capture how researchers operationalised the concept: (1) the daily realities of structural vulnerability and (2) the upstream drivers of structural vulnerability.

##### 4.3.2.1 Daily Realities of Structural Vulnerability

Several studies examined everyday conditions, such as economic insecurity, housing instability, and early independence from familial support, that, shaped care‐seeking, chronic disease self‐management and health‐related decision‐making (Brantley, Footer, et al. 2017; Manser et al. 2024; Zhen‐Duan et al. 2022). Other studies explored place‐based inequities, including neighbourhood disinvestment, occupational segregation, policing patterns and environmental hazards. These were acknowledged as structural constraints contributing to heightened risks of overdose, injury, sexual health challenges and threats to personal safety, all of which participants navigated in the course of everyday life (Arnold et al. 2021; Chang et al. 2019; Friedman et al. 2019, 2021; Haas et al. 2018; Holmes 2011; McKenna 2014; Organista et al. 2013; Worby et al. 2014).

##### 4.3.2.2 Upstream Drivers of Structural Vulnerability

Qualitative studies used structural vulnerability to trace how upstream forces, including institutional policies, historical injustices and dominant ideologies, limit access to fundamental rights, care and physical safety. Drawing on participant observation, in‐depth interviews or multiple qualitative methods, researchers explored how structural processes, such as racialization, gender discrimination, stigmatising medical encounters, and contested citizenship, shaped individuals' exposures to harm, COVID‐19 testing, health behaviours and access to care (Arnold et al. 2024; Collins et al. 2024; Holmes 2011; Kohut et al. 2024; Yang et al. 2014; Friedman et al. 2019; Tulimiero et al. 2021; Westbrook 2024; Young et al. 2022).

## 5 Discussion

This review synthesises how structural vulnerability has been defined and operationalised across empirical health studies. Across methodological approaches, definitions remained consistent with the concept's critical origins (Bourgois et al. 2017; Quesada et al. 2011); however, the strategies for operationalising the concept varied. Quantitative studies frequently measured individual‐level factors, whereas qualitative and mixed methods studies more explicitly focused on structural determinants.

Across the literature reviewed, structural vulnerability was understood through two interrelated dimensions—as a lived reality (a social positionality) and as a critical framework for understanding how that reality is produced. As a positionality, structural vulnerability is characterised by constrained resilience, limited agency, and imposed risks rooted in systemic discrimination and reinforced by a social hierarchy. As a critical framework, it prompts analysis of the social, economic and political mechanisms that produce, organise and sustain contexts of suffering and disadvantage.

While definitional agreement was strong, operationalization diverged. The extent to which studies sought to critically illuminate or intervene upon structural forces is where this was most evident. Quantitative studies contributed tools for identifying population‐level patterns but often reduced structural vulnerability to individual‐level factors. This nominal use missed the concept's underlying premise that health inequities are produced through broader systems of power, inequality and exclusion. Future operationalizations of structural vulnerability could be strengthened in a number of ways, such as incorporating analysis of historical records and policies, such as patterns of racial segregation, and economic resource allocation (Bailey et al. 2017), or through developing multi‐level models that examine how structural contexts interact with individual exposures and outcomes.

In contrast, the qualitative and mixed methods studies in this review more consistently operationalised structural vulnerability in alignment with its critical intent—to uncover the structural factors that disproportionately burden marginalised populations (Bourgois et al. 2017; Quesada et al. 2011). These studies foregrounded the participants' lived experiences as critiques of structural forces such as labour exploitation, criminalization, systemic racism and gentrification. With structural vulnerability as a lens, they illuminated how exclusionary ideologies, such as citizenship‐based notions of worthiness, shaped the contexts in which people were living and working and their health experiences (e.g., Arnold et al. 2021, 2024; Holmes 2011; Tulimiero et al. 2021; Westbrook 2024; Young et al. 2022). A common limitation, however, was that few extended their insights to the design or implementation of structural interventions, or to identifying concrete actions to reduce structural vulnerability and influence outcomes (e.g., Tulimiero et al. 2021).

This examination and critique of qualitative and quantitative literature is not intended to position one methodology over the other, but to acknowledge their distinct strengths and complementary opportunities for operationalising structural vulnerability. Maintaining the concept's critical perspective requires resisting the conflation of structural vulnerability with adjacent concepts such as ‘social vulnerability’, or with individual‐level analysis of conventional socioeconomic measures (Quesada et al. 2011; Bourgois et al. 2017; Rao et al. 2023). Building on the shared understanding of the concept's definitional domains, it follows that approaches to operationalization must attend to the systems, structures, and ideologies that produce risk and constrain agency. Why and how individuals or groups come to have particular experiences, and live in certain contexts, is key. Maintaining *structural* inquiry is therefore integral to operationalising structural vulnerability.

To distinguish structural inquiry from individual inquiry requires attending to the focus of the analysis. Given that individuals are situated within broader contexts, structural and individual inquiries are inherently related. However, they differ in where emphasis is placed. Centering experiences articulated from unique standpoints (Collins 2022), is a key strategy for revealing the structures—such as power dynamics, economic policies, structural racism, gender inequality and ableism—that exert influence. When lived experience is leveraged for the critical interrogation of the structural contexts shaping lives and possibilities, deeper insights emerge (Bailey et al. 2017; Bourgois et al. 2017; Collins 2022). Qualitative tools such as the Structural Vulnerability Assessment Tool (SVAT) and its quantitative adaptations, or any rigorously constructed, validated measure, hold particular promise for structural inquiry when deployed to identify, measure or intervene upon structural determinants. Conversely, approaches that generate individual‐level risk profiles, without a related structural analysis, miss the opportunity for structural inquiry. This distinction lies in the difference between asking, for example, *why* particular groups are living in poverty, as opposed to merely acknowledging *that* they are. Structural vulnerability operating as structural inquiry is therefore not bound by any particular methodology, but by the orientation of the research towards analysing systems of power and inequality.

Within the primary aim of structural inquiry, structural vulnerability opens up opportunities across methodological approaches. Qualitative methods—such as narrative inquiry, oral history and ethnography—that centre participants' epistemologies are fundamental to revealing how oppressive contexts are influencing health outcomes. Uncovering the nuanced ways structural forces are felt and navigated can guide the development of targeted structural interventions. Likewise, quantitative analyses can ‘tell the story’ of social, political and economic contexts, leveraging advanced statistical techniques—including predictive modelling—to expose the effects of systemic practices undermining population health and well‐being. Collectively, diverse methodological approaches offer distinct yet complementary tools and a host of possibilities for operationalising the concept.

In addition to the methodological considerations, there remain opportunities for future applications of structural vulnerability across a wider range of geographies and populations. The geographic location of the studies in this review was concentrated in a few U.S. regions, and notably the Mid‐Atlantic region (eleven studies were conducted in Baltimore, MD; Table 1). Similarly, the populations studied included marginalised groups, with a notable representation of cisgender women who engaged in sex work, Latino migrant labourers, transgender and gender‐diverse individuals and people who use substances. To a lesser degree, studies focused on child farmworkers and unstably housed individuals. There remains a significant opportunity to apply the concept of structural vulnerability through research partnerships with a broader range of communities navigating contexts of risk and disadvantage, such as birthing people, disabled individuals, incarcerated and formerly incarcerated people, those impacted by community violence, Indigenous communities and groups affected by environmental injustice.

Despite important contributions, the current body of empirical health research applying structural vulnerability remains narrow in scope in the health outcomes examined, and the temporal scale of inquiry. Existing work has centred on infectious disease, substance use, and mental health while outcomes like chronic illness, maternal and reproductive health, preventive care and aging remain underexplored. Methodologically, cross‐sectional designs predominate, limiting the ability to trace how structural harm accumulates and unfolds over time. To date, no study has examined structural vulnerability with the longitudinal depth necessary to examine it as an intergenerational phenomenon. Finally, few studies have examined the ways individuals, families, and communities exhibit, or cultivate, resilience, potentially reinforcing a deficit‐oriented scope of knowledge that has been common in health disparity research (Márquez‐Magaña 2024).

In addition to widening the scope of inquiry, meaningfully integrating structural vulnerability into research requires frameworks for structural awareness, reflexivity and ethical engagement. Structural competency is increasingly integrated into health training curricula to equip learners and practitioners to recognise and analyse systemic barriers to health and well‐being (Metzl and Hansen 2014). Structural humility calls for ongoing reflection on power dynamics, the limitations of one's own knowledge, and the positionality of providers or researchers in relation to the communities they serve or partner with for research (Samarasekera 2022). Together, structural competency and structural humility offer complementary frameworks for fostering ethical research approaches that aim to reveal structural forces and position them as both measurable and open to critique and transformation (Márquez‐Magaña 2024; Metzl and Hansen 2014).

The shift in perspective from an individual‐level focus to a structural analysis constitutes the central contribution of the concept of structural vulnerability. However, how researchers operationalise this shift in practice depends heavily on their epistemological stance. That is, their underlying assumptions around knowledge production, lived experience, and the nature of evidence will impact how the concept is taken up and applied. Epistemological orientation shapes not only the methodologies employed but also the capacity of research to expose, and ultimately to support the transformation of, the structural forces influencing health outcomes (Bourgois et al. 2017; Collins 2022).

The findings from this review, and particularly the patterns and limitations in how the concept has been operationalised, reveal an opportunity to more clearly articulate the concept's philosophical grounding in a relational ontology. A relational ontology posits that the nature of entities, whether individuals, objects, or concepts, is constituted through their relationships with other entities and contexts (Benjamin 2015). From this perspective, structural vulnerability is a social phenomenon that is emergent and dynamic; it is not a fixed attribute of individuals or groups, but arises through the entanglement of macro‐level forces (e.g., policy, institutional power and social hierarchies) with everyday micro‐level lived realities. This framing highlights how power operates relationally to disempower, shaping the embodied experiences of social inequality that often manifest as health inequities.

This review is subject to several limitations. First, the search strategy may not have captured all relevant studies applying the concept of structural vulnerability, potentially omitting important perspectives. Second, it focused exclusively on literature that explicitly invoked the concept of structural vulnerability, potentially omitting studies with similar theoretical and ethical foundations that may have offered valuable insights. Third, given the vast diversity of marginalised and disadvantaged populations in the U.S., the reviewed literature does not fully reflect the range of contexts in which structural vulnerability is produced and experienced. Despite these limitations, this synthesis provides a foundation for future applications of structural vulnerability in equity‐centred health research, aligned with the concept's critical origins.

## 6 Conclusion

Structural vulnerability is a relationally produced phenomenon with significant impacts on health. People are not inherently structurally vulnerable; rather, vulnerability emerges in response to systems of inequality. If vulnerability is structurally produced, it can—and must—be structurally undone. Addressing structural determinants of health inequities requires inquiry and interventions that confront harmful systems and build just alternatives. This demands conceptual rigour, ethical clarity and critical reflexivity, but above all sustained action towards systemic change, grounded in accountability and guided by the pursuit of health justice.

## Conflicts of Interest

The authors declare no conflicts of interest.

## Supporting information


**Table S1:** jan70408‐sup‐0001‐TableS1.pdf.

**Table S2:** jan70408‐sup‐0002‐TableS2.pdf.

**Table S3:** jan70408‐sup‐0003‐TableS3.pdf.

## Data Availability

The data that supports the findings of this study are available in the Supporting Information of this article.
