# Supplementary material for: Structural Vulnerability in Health Research: A Systematic Mixed Studies Review
Source: J Adv Nurs. 2025 Dec 1;82(7):7104–25. doi: 10.1111/jan.70408 (PMC13267471; doi:10.1111/jan.70408)
Supplement: Supplementary file 1 — Table S1: jan70408‐sup‐0001‐TableS1.pdf. [file JAN-82-7104-s002.pdf]

Supplementary Table 1. Literature search strategy

Pub Med - 266 Citations retrieved
Search: (“structural* vulnerab*”[Title]) OR (“structural* vulnerab*” [Title/Abstract] AND health [Title/Abstract] Filters: English, Humans
Embase - 132 Citations retrieved
('structural* vulnerab*’:ti OR ('structural* vulnerab*’:ab AND health:ab)) AND [embase]/lim AND [humans]/lim AND [english]/lim AND ‘human’/de AND ‘article’/it
Scopus - 103 Citations retrieved
Title: “structural* vulnerab*” OR Abstract: “structural* vulnerab*” AND health Limits: Keywords - Structural vulnerability; Subject area – Medicine, Social Sciences, Arts and Humanities, Psychology, Multidisciplinary, Agricultural and Biological Sciences, Nursing, Health Professions; Country – United States; Language – English
CINAHL - 74 Citations retrieved
Search: TI “structural* vulnerab*” OR AB (“structural* vulnerab* AND health) Limits: USA; English language
