# Supplementary material for: Structural Vulnerability in Health Research: A Systematic Mixed Studies Review
Source: J Adv Nurs. 2025 Dec 1;82(7):7104–25. doi: 10.1111/jan.70408 (PMC13267471; doi:10.1111/jan.70408)
Supplement: Supplementary file 2 — Table S2: jan70408‐sup‐0002‐TableS2.pdf. [file JAN-82-7104-s003.pdf]

Supplementary Table 2

Quantitative Studies

Citation	Purpose	Sample & Setting	Design & analytic strategy	Definition of Structural vulnerability	Operationalization of Structural Vulnerability	Health Outcomes/Related Factors	Key Findings	Limitations
Beckham et al. (2022)	To describe patterns of substance use and associations with HIV-related risks and structural vulnerabilities among cisgender women engaged in street-based transactional sex.	244 cisgender women who engage in street-based transactional sex<br><br>Mean age = 35.8 years<br><br>32.8% Black, Indigenous, and People of Color and Hispanic<br>67.2% Non-Hispanic White<br><br>98% reported non-injection drug use<br>68% reported injection drug use<br><br>Recruited from locations known for sex work activities<br><br>April 2016 – August 2017<br><br>Baltimore, MD	Cross sectional, analytic<br><br>Data collected via survey<br><br>Secondary data analysis of the Sex Workers and Police Promoting Health in Risky Environments (SAPPHIRE) study a prospective cohort study<br><br>Partnership with a community advisory board<br><br>Latent class analysis (LCA) to identify profiles of polysubstance use.	"The environmental and structural factors that place women 'at risk of being at risk' beyond looking at individual behavior alone."	Structural vulnerability was operationalized using three key indicators measured as outcome (dependent) variables<br><br>Housing insecurity<br>Past or current homelessness<br><br>Food insecurity<br>Going to bed hungry at least 1x/week<br><br>Obtaining syringes from a syringe service program (SSP)<br>Only getting syringes from an SSP	HIV-Related Risks<br>condomless sex<br><br>Exposure to client violence<br>Physical or Sexual<br><br>Measured as outcome/dependent variables<br><br>———<br><br>Substance use over past 3 months<br><br>Type of drug and route incorporated as the latent class indicators	A latent class analysis identified three substance use profiles: Heroin/Cocaine (48.4%), Poly-opioid (21.3%), and Poly-route, Polysubstance use (30.3%). High rates of housing (53–68%) and food insecurity (45–66%) were observed across all classes, though differences were not statistically significant.<br><br>Poly-route participants—more likely to be young, White women—had the highest SSP use and lowest HIV risk behaviors.<br><br>In contrast, Poly-opioid (more likely BIPOC) showed lower SSP engagement.<br><br>The findings suggested that Baltimore's SSPs may be under-serving racial and ethnic minority women.	Cross-sectional design limiting ability to establish direction of causality.<br><br>Power analysis not reported; the study may not be adequately powered to detect statistically different outcomes among the latent classes.

Citation	Purpose	Sample & Setting	Design & analytic strategy	Definition of Structural vulnerability	Operationalization of Structural Vulnerability	Health Outcomes/Related Factors	Key Findings	Limitations
Brantley et al. (2017b)	To profile experiences of structural vulnerability by identifying distinct patterns of co occurring social and economic disadvantage.<br><br>Using indicators of structural vulnerability related to housing, finances, education, and arrest, latent class analysis was used to investigate how different indicators cluster together and are associated with drug use and sexual risk behavior.	117 women working in an exotic dance club (EDC)<br><br>Median age = 24 years<br><br>35% White<br>65% Non-white<br><br>Purposively sampled from 22 EDC environments classified as High (n=12) or Low (n=10) HIV risk based on drug and sex risk profiles established from prior data.<br>68% of sample worked in high risk EDC.<br><br>48% worked in EDC for 4 months or less<br><br>May – October 2014<br><br>Baltimore, MD	Cross-sectional, analytic; multi-level<br><br>Data collected via survey<br><br>Latent Class Analysis (LCA)<br>Bivariate latent class regression	Structural vulnerability is a concept that "relates societal contexts to an individual's ability to control health outcomes."<br>Structural vulnerability occurs "as a result of policies and systems that create disadvantage based on a set of characteristics," and which, at an individual level, "surfaces as an accumulation of multiple social and economic disadvantages."	Structural vulnerability was operationalized through a series of binary indicators reflecting instability across key life domains.<br><br>These variables were coded as binary (Yes/No) and combined to form a composite latent variable representing overall structural vulnerability in the analysis.<br><br>Housing<br>Homelessness<br>Temporary housing<br>Moving more than twice<br><br>Financial insecurity Being in debt<br>Being behind in rent<br>Borrowed money for rent in past 6 months<br><br>Limited education<br>Did not graduate high school<br>High school diploma or GED<br>Exposure to college but dropped out<br><br>Experiences with the Criminal Justice system<br>History of at least one arrest during adulthood	HIV/STI Risk Behaviors<br>Drug use; Sexual risk<br><br>EDC HIV/STI Risk<br>High vs. low<br><br>Self-reported Health<br>Good/excellent<br><br>History of abuse<br>Intimate partner violence (IPV)<br>Childhood sexual or physical violence<br><br>Depression<br>CES-D* ≥ 16<br><br>Bacterial STI<br>Gonorrhea or Chlamydia infection<br><br>*Center for Epidemiological Studies – Depression Scale	A two-class latent class analysis identified a Low Vulnerability group (68%) and a High Vulnerability group (32%). The High Vulnerability group had high probabilities of housing and financial insecurity, with moderate levels of limited education and arrest history.<br><br>Age and race did not differ between groups, but experiences of IPV and depression increased the likelihood of high vulnerability.<br><br>Women in the High Vulnerability group were more likely to report HIV/STI risk behaviors, including multiple sex partners, sex exchange, and recent illicit drug use.	Cross-sectional design limiting ability to establish direction of causality.<br><br>No power analysis reported. Adequacy of the sample size for analysis is unclear<br><br>Sample size restricted options for statistical analysis<br><br>Race categorized as white vs. non-white; racial categories within the non white group (65%) are not reported<br><br>Imprecise odds ratios (wide CIs) for outcome of interest (drug use); a large portion (68%) of the sample recruited from high-risk EDCs, reducing variability in risk exposure; limiting the generalizability of the findings.

Citation	Purpose	Sample & Setting	Design & analytic strategy	Definition of Structural vulnerability	Operationalization of Structural Vulnerability	Health Outcomes/Related Factors	Key Findings	Limitations
Footer et al. (2020)	To analyze how structural factors, such as food insecurity, and homelessness manifest in the present day lives of those who traded sex under 18 years of age versus later in life, in addition to exploring the relationship of homelessness and food insecurity to female sex workers current HIV-risk behaviors.	250 cis-gender women involved in street-based sex work<br><br>Mean age = 35.7 years (range 18-61 years)<br><br>66.4% White<br>22.8% Black<br>10.8% Hispanic & other<br><br>Mean age of entry into the sex trade was 24.7 years (range 11–61)<br>21.2% of women entered < 18 years<br>78.8% entered at age 18 or older<br><br>Recruited from locations known for sex work activities<br><br>April 2016 – August 2017<br><br>Baltimore, MD	Cross-sectional, analytic<br><br>Data collected via survey and biological specimens<br><br>Secondary data analysis of the Sex Workers and Police Promoting Health in Risky Environments (SAPPHIRE) study a prospective cohort study<br><br>Partnership with a community advisory board<br><br>Logistic regression<br>Bivariate<br>Multivariable	"An important explanatory framework that can help articulate how a persons' positionality in a hierarchical social order characterized by intermediate factors of social and economic disadvantage can translate into micro-level individual level hardship, which in turn shape more proximal HIV-risk factors."	Structural vulnerability was operationalized using both outcome and covariate measures reflecting material deprivation and systemic exclusion.<br><br>Primary outcome variables included recent homelessness (experienced in the past three months) and food insecurity (frequency of going to bed hungry in the past three months).<br><br>Additional indicators were measured as covariates and included:<br><br>Limited education<br>Not graduating high school vs. graduating high school or obtaining GED<br><br>Current unemployment<br><br>No monthly savings<br>Over the last 3 months<br><br>Arrest history<br><br>Healthcare access<br>Insurance status, no doctor visit in past 12 months, visit to the emergency department in past 3 months	Proximal drivers of HIV risk<br>within past 3 months<br>STI infection<br>Inconsistent condom use<br>> 30 sex clients<br>Daily heroin use<br><br>Incorporated as predictors in regression analysis	Findings revealed that 20.8% of women who entered the sex trade before age 18 were forced into it, compared to 4.6% of those entering at 18 or older.<br><br>Many entered for survival: 35.6% for basic necessities and 17.2% to support their families. Over 60% experienced hunger and homelessness.<br><br>Early entry and daily heroin use were linked to higher odds of homelessness, while early entry alone was associated with greater food insecurity.<br><br>Longer time in the sex trade was tied to reduced homelessness. Recent homelessness increased STI risk, and recent food insecurity was associated with higher odds of client violence and HIV risk.	Cross-sectional design limiting ability to establish direction of causality.<br><br>Recall bias is present with self-reported data, particularly for participants who have been involved in sex trading for longer.<br><br>Social desirability bias may have impacted participants' reporting their age of entry to sex trading<br><br>Small sample size of women who entered as minors impacted the precision of the statistical analysis<br><br>Structural indicators in analysis were selected for their "salience as independent health indicators" without rationale for why other SV indicators were not explored.

Citation	Purpose	Sample & Setting	Design & analytic strategy	Definition of Structural vulnerability	Operationalization of Structural Vulnerability	Health Outcomes/Related Factors	Key Findings	Limitations
Glick et al. (2020)	To examine the differences in structural vulnerabilities and HIV risk drivers between sexual minority women, by identity and behavior, and their heterosexual counterparts among street-based female sex workers.	247 cis-gender women involved in street-based sex work<br><br>Mean age = 35.7 years<br><br>34% People of color<br>66% White<br><br>25.5% self-identified as a sexual minority (SM) by identity<br><br>8.5% reported SM behavior<br><br>51.8% had been engaging in sex work for more than 5 years<br><br>Recruited from locations known for sex work activities<br><br>April 2016 – August 2017<br><br>Baltimore, MD	Cross-sectional, analytic<br><br>Data collected via survey<br><br>Secondary data analysis of the Sex Workers and Police Promoting Health in Risky Environments (SAPPHIRE) study a prospective cohort study<br><br>Partnership with a community advisory board<br><br>Pearson's chi-square<br>Simple linear regression<br>Multivariable logistic regression	Structural vulnerability reflects "societal level factors that constrain an individual's agency particularly related to health outcomes."	Structural vulnerability was operationalized using a set of binary and continuous indicators reflecting cumulative exposure to systemic and interpersonal adversity.<br><br>Homelessness<br>Over past 3 months<br><br>Lifetime arrest<br><br>Food insecurity<br>Measurement approach not reported<br><br>Childhood abuse<br>Sexual or physical<br><br>Intimate partner violence (IPV)Sexual or physical<br><br>HIV<br>Positive test<br><br>Substance use<br>Injection drug use (IDU)<br>Drug use<br>Binge drinking<br><br>Sex work characteristics<br>Minor at sex work entry<br>Ever had pimp/manager<br>Years in sex work	HIV risk drivers<br>Specific indicators measured as HIV risk drivers are not made clear<br><br>Measured as outcome/dependent variables	In the total sample, high levels of structural vulnerability were reported: 62.8% had experienced homelessness, 82.2% had been arrested, and over half were food insecure.<br><br>Childhood abuse, IPV, and substance use were also common. Among participants, 21.5% entered sex work as minors, and 9.3% had a pimp or manager.<br><br>Sexual minority female sex workers (SM-FSW) reported higher rates of most structural vulnerabilities compared to heterosexual FSW, with significant differences in age, homelessness, and physical IPV.<br><br>SM-FSW classified by behavior had higher odds of binge drinking, homelessness, and minor entry, while FSW who self-identified as SM had higher odds of IDU and physical IPV.	Cross-sectional design limiting ability establish direction of causality.<br><br>Authors utilized a binary race variable; no assessment of the differences among people of color.<br><br>Analysis only included cisgender women which excluded the possibility of exploring the risk profiles of gender diverse individuals.<br><br>Measurements of depression and PTSD are reported, but instruments and their associated validity and reliability for use with the study sample are not reported.

Citation	Purpose	Sample & Setting	Design & analytic strategy	Definition of Structural vulnerability	Operationalization of Structural Vulnerability	Health Outcomes/Related Factors	Key Findings	Limitations
James & Horne (2024)	To examine if internalized racism explained the relationship between healthcare access barriers and health attitudes.	780 Black American Adults<br><br>Mean age = 37.7<br><br>42.2% Men<br>57.6% Women<br><br>National online survey	Cross-sectional, analytic<br><br>Data collected via electronic survey<br><br>Effect analysis<br>Chi-square<br>Pearson's correlation	"The structural vulnerability framework is used to understand how social structures, institutions and policies contribute to the vulnerability of marginalized or disadvantaged populations"	SV operationalized as an analytical framework (integrated with Internalized Racism Framework) to inform their investigation of the relationships among structural barriers to healthcare access, internalized racism, and attitudes toward healthcare seeking across four health domains.	Health care access barriers<br>Health attitudes	Structural barriers often co-occur across domains, suggesting the need for holistic healthcare reform that addresses insurance, affordability, transportation, and discrimination simultaneously.<br><br>Attitudes toward seeking care varied by domain, with the most positive attitudes toward vision care and the least toward mental health, yet were positively correlated across domains.<br><br>Internalized racism significantly mediated the relationship between structural barriers and negative health-seeking attitudes in medical, mental, and vision care, but not in dental care, where cost and fear-based avoidance may play a larger role.	Cross-sectional design limiting ability establish direction of causality.<br><br>All data on healthcare barriers were based on participants' self-reports, which may introduce recall bias or subjective misperceptions that don't reflect objective realities.<br><br>The authors noted the measures for attitudes toward dental and vision care lack formal validation. The scales measuring attitude toward seeking psychological and dental care showed low internal consistency in this sample.

Citation	Purpose	Sample & Setting	Design & analytic strategy	Definition of Structural vulnerability	Operationalization of Structural Vulnerability	Health Outcomes/Related Factors	Key Findings	Limitations
Jegede et al. (2021)	To assess perceived structural vulnerability, perceived barriers to access care and anticipated discrimination among African American patients currently in inpatient treatment for substance use disorder (SUD).	58 African American patients receiving inpatient treatment for a SUD<br><br>Mean age = 49.6 years<br><br>79.3% male<br>20.7% female<br><br>Urine toxicology screen<br>42.8% alcohol<br>27% cocaine<br>17.4% opioids, benzodiazepines and other substances<br>12.7% cannabis<br><br>44.8% with a psychiatric diagnosis<br><br>A community inpatient substance use treatment setting<br><br>September – November 2019<br><br>Brooklyn, NY	Cross-sectional, descriptive<br><br>Data collected via survey<br><br>Descriptive and frequency statistics	The "condition of an imposed risk of negative health outcomes, for an individual or population, 'through their interface with socioeconomic, political and cultural/normative hierarchies'. Patients present structurally vulnerable when these aforementioned factors interfere with their abilities to access or benefit from reasonable care."	Structural vulnerability was operationalized using the Structural Vulnerability Assessment Tool (SVAT), which captures key domains linked to systemic disadvantage, including financial security, housing status, exposure to risk environments, food access, social network support, legal status, education, and experiences of discrimination.<br><br>Each domain was measured using binary (Yes/No) indicators, allowing for the identification of specific structural vulnerabilities experienced by participants.	Perceived barriers to access care<br>Barriers to Access Care Evaluation (BACE-3)<br><br>BACE-3 Stigma subscale<br><br>Anticipated discrimination<br>Questionnaire on Anticipated Discrimination (QUAD)<br><br>Mean scores calculated	83% reported a lack of financial security<br><br>71% reported not having a safe residence<br><br>60% reported not feeling safe and healthy in places where they spend their time each day<br><br>76% reported experiencing discrimination	Cross-sectional design limiting ability to establish direction of causality.<br><br>Limited sample size, and limited to inpatient participants – limited generalizability<br><br>Limited statistical analyses<br><br>Adaptation of the SVAT removed the reflexivity probe intended for the provider found in the discrimination domain

Citation	Purpose	Sample & Setting	Design & analytic strategy	Definition of Structural vulnerability	Operationalization of Structural Vulnerability	Health Outcomes/Related Factors	Key Findings	Limitations
King et al. (2023a)	To describe sociodemographic patterns in self-reported structural needs and unmet structural needs, examine the relationship between structural needs, mental health, and substance use outcomes among trans young adults.	215 transgender and nonbinary young adults<br><br>Mean age = 24.4 years (range 18 – 29)<br><br>37.4% White<br>18.2% Latinx<br>17.3% Asian/Pacific Islander<br>15.4% Multiracial/ethnic<br>8.9% Black<br>1.9% Native American<br><br>52.1% non-binary+<br>29.3% women/feminine<br>18.6% men/masculine<br><br>87.4% US born<br><br>2019 – 2021<br>(months not provided)<br><br>San Francisco Bay Area, California	Cross-sectional, analytic<br><br>Data collected via survey<br><br>Secondary data analysis of the quantitative baseline data from the Phoenix Study, a mixed methods longitudinal investigation of substance use behaviors<br><br>Simple linear regression<br>Multivariable logistic regression<br>Mediation analysis	"Structural vulnerabilities are experiences that threaten an individuals' survival, such as limited food access, housing deprivation, and interpersonal violence."	Structural vulnerability was measured through self-reported structural needs experienced over the past six months, including access to housing, food assistance, job training, disability benefits, health insurance, utility payment assistance, unemployment benefits, legal assistance, and crisis intervention.<br><br>These indicators were used to create two separate scales:<br>Structural Needs Scale: Count of structural resources needed (range: 0–9)<br>Unmet Structural Needs Scale: Count of structural needs that went unmet (range: 0–9)<br><br>Both scales were treated as continuous variables and incorporated as predictors in regression models to examine associations with health and behavioral outcomes.	Mental health<br>Suicidal ideation<br>Depressive symptoms<br>Gender related PTSD<br><br>Substance use<br>Alcohol<br>Tobacco<br>Illicit drugs<br><br>Measured as outcome/dependent variables	Participants commonly reported structural needs for health insurance (45.1%), food assistance (43.7%), and housing (34%), with unmet needs most often in legal aid (19.1%) and unemployment benefits (16.7%).<br><br>On average, individuals had 2.4 structural needs and 1.2 unmet needs. Each additional structural need was linked to increased odds of daily marijuana use (29%), suicidal ideation (24%), and higher depressive symptoms.<br><br>Unmet needs were also associated with greater marijuana use and depression. Both structural and unmet needs fully mediated the relationship between nontraditional income sources and depressive symptoms.	Cross-sectional design limiting ability to establish direction of causality.<br><br>Limited to participants who can communicate in English; and convenience sampling method, limits the generalizability<br><br>Risk of social desirability bias and recall bias with self-report of substance use and mental health history

King et al. (2023a). Structural Needs, Substance Use, and Mental Health Among Transgender and Nonbinary Young Adults in the San Francisco Bay Area: Findings from the Phoenix Study.

Citation	Purpose	Sample & Setting	Design & analytic strategy	Definition of Structural vulnerability	Operationalization of Structural Vulnerability	Health Outcomes/Related Factors	Key Findings	Limitations
King et al. (2023b)	To demonstrate the utility of using a structural vulnerability framework to understand distributions of adverse health outcomes among trans populations.<br>Examine patterns of structural vulnerability and explore the relationship between structural vulnerability and mental health and substance use outcomes.	60 participants that self-identified as transgender women of color<br><br>Mean age = 29 years (range 18 – 54)<br><br>83.3% Black/African American<br>16.7% Latina<br><br>January – September 2020<br><br>Detroit, MI	Cross-sectional, analytic<br><br>Data collected via survey<br><br>Secondary analysis of the data from a needs assessment survey developed and implemented by the Love Her Collective; Researchers and community members developed the survey collaboratively<br><br>Logistic regression<br>Latent class analysis (LCA)	"The concept of structural vulnerability explains how systems of oppression drive health inequities by reducing access to survival resources for marginalized populations."	Structural vulnerability was operationalized using the Structural Vulnerability Assessment Tool (SVAT)<br><br>These SV indicators were used both as predictors in regression analyses and as latent class indicators to identify distinct subgroups based on patterns of structural vulnerability.<br><br>Financial security<br>Enough money to live<br>Monthly income<br>Residence<br>Own or rent home<br>Financial worry about housing<br>Risk environments<br>Exposure to IPV<br>Anti-trans victimization<br>Food access<br>Missed meals/not enough money for food<br>Social network<br>Social rejection<br>Legal status<br>Legal gender affirmation needs met<br>Education<br>Highest level of schooling<br>Discrimination<br>Anti-trans discrimination	Mental health<br>PTSD symptoms<br>Post Traumatic Stress Checklist (PCL-2)<br><br>Anxiety symptoms<br>Generalized Anxiety Disorder scale (GAD-7)<br><br>Suicidal ideation<br>Single item questionnaire<br><br>Substance use<br>Tobacco, marijuana, and use of other substances - "club drugs" - over the past 3 months<br><br>Alcohol use<br>Over the past year<br><br>Measured as outcome/dependent variables	Most structural vulnerability indicators were linked to poorer mental health outcomes.<br><br>Financial worry was associated with increased marijuana use, and IPV was linked to higher alcohol use.<br><br>A latent class analysis identified three subgroups:<br>Low Vulnerability (39%)<br>High Economic Vulnerability (32%)<br>Complex Multi-Vulnerability (29%)<br><br>Compared to other groups, Low Vulnerability was associated with lower odds of PTSD, while High Economic Vulnerability was linked to increased marijuana use.<br><br>The Complex Multi-Vulnerability group showed the highest risk for PTSD, anxiety, suicidal ideation, and club drug use.<br><br>These associations were more clearly observed through latent class analysis than through individual indicator analysis.	Cross-sectional design limiting ability to establish direction of causality.<br><br>Small sample size, study conducted during the COVID-19 pandemic, limiting generalizability<br><br>Limited constructs of structural vulnerability relevant to the study population<br><br>Study measures did not assess additional axes of oppression such as racism, ageism, or ableism.

King et al. (2023b). Structural vulnerability as a conceptual framework for transgender health research: findings from a community needs assessment of transgender women of colour in Detroit.

Citation	Purpose	Sample & Setting	Design & analytic strategy	Definition of Structural vulnerability	Operationalization of Structural Vulnerability	Health Outcomes/Related Factors	Key Findings	Limitations
Lim et al. (2019)	To identify correlates – structural vulnerability and health factors - of severe food insecurity among street-based FSW in Baltimore, Maryland.	249 street-based female sex workers<br><br>Mean age = 35.7 years<br><br>66.3% White<br>22.9% Black<br>10.8% Hispanic<br><br>51.8% Sex work >5 years<br>66/3% Daily sex work<br><br>April 2016 – August 2017<br><br>Baltimore, MD	Cross-sectional, analytic<br><br>Data collected via survey and biological specimens<br><br>Secondary data analysis of the Sex Workers and Police Promoting Health in Risky Environments (SAPPHIRE) study a prospective cohort study<br><br>Partnership with a community advisory board<br><br>Bivariate analyses<br>Multivariable logistic regression	Not specified	Structural vulnerability was operationalized using five binary indicators reflecting cumulative disadvantage.<br><br>These indicators were incorporated as predictors in regression analyses to examine their association with health and behavioral outcomes.:<br>recent homelessness (past 3 months<br>limited education (not completing high school)<br>lifetime arrest<br>lifetime incarceration (incarcerated for more than two days)<br>receipt of any public benefit.	Food insecurity<br>Single-item question; Over past 3 months<br>Binary: severe or not severe<br><br>Measured as outcome/dependent variable<br><br>---<br><br>Depression symptoms<br>CESD-10; ≥15 = moderate to severe symptoms<br><br>PTSD symptoms<br>PCL-5; score subcategorization as binary yes/no<br><br>Substance use<br>Past 3 months; various measures<br><br>Violence victimization<br>Childhood (lifetime) or adulthood (over past 3 months<br><br>HIV/STI infection<br>Yes/no<br><br>Incorporated as predictors in regression analysis	Over 26% of participants experienced severe food insecurity—significantly higher than the general U.S. population.<br><br>The majority reported recent homelessness (62%), less than a high school education (53%), lifetime arrest (82%), and incarceration (70%).<br><br>Over half (53.4%) cited the need to buy food as a reason for engaging in sex work. Compared to those with no or moderate food insecurity, women with severe food insecurity were more likely to be younger, sell sex daily, be homeless, and less likely to receive public benefits.<br><br>Overall, the sample showed high levels of overlapping vulnerabilities, including housing instability, incarceration, violence, mental health symptoms, and substance use.	Cross-sectional design limiting ability to establish direction of causality.<br><br>A single item to measure individual-level food insecurity, may not have adequately captured the complexities associated with food insecurity.<br><br>Validity and reliability of measures not reported

Citation	Purpose	Sample & Setting	Design & analytic strategy	Definition of Structural vulnerability	Operationalization of Structural Vulnerability	Health Outcomes/Related Factors	Key Findings	Limitations
Organista et al. (2019)	To test a model of hypothesized pathways between working and living conditions and multiple forms of psychological distress.	344 Latino migrant day laborers (LMDLs)<br><br>Approximately 50% Mexican and Central American<br><br>92% were undocumented<br><br>Mean age = 40 years<br><br>Mean years in the US = 12.5<br>Mean weekly wages = $232<br>50% considered their pay "Bad" or "Awful"<br>14.5% considered their pay "Good"<br>"Most" consider their pay to be "less than fair"<br><br>Sampled from the six busiest LMDL work pick-up sites in San Francisco and Berkeley<br><br>Dates not specified<br><br>San Francisco and Berkeley, CA	Cross-sectional, analytic<br><br>Data collected via surveys<br><br>Structural equation modeling (SEM)<br>Each of the four psychological outcome variables were modeled as separate latent indicators	"A positionality of LMDLs in the United States as characterized by difficult living and working conditions produced and reproduced by particular sets of global economic, political, social, and cultural factors."	Structural vulnerability was operationalized through two independent latent variables capturing dimensions of working and living conditions.<br><br>Working conditions<br>Earnings per week<br>Proportion of days and hours worked during past week<br><br>Living conditions<br>Level of trust for roommates<br>How well they get along with roommates<br>How much where they live feels like home	Depression<br>Center for Epidemiological Studies-Depression Scale (CES-D)<br><br>*Desesperación* *<br>Scale of *Desesperación* (desperation)<br><br>Anxiety<br>Generalized Anxiety Disorder screening instrument (GAD-7)<br><br>Alcohol use<br>The 10-item Alcohol Use Disorder Identification Test (AUDIT)<br><br>Measured as outcome/dependent variables<br><br>*a culture-based idiom of distress frequently expressed by LMDLs	SEM revealed pathways linking challenging living and working conditions to each other – indicating that as one improves, so does the other.<br><br>Direct paths in the model also linked both working and living conditions to *desesperación* and depression, and link living conditions to alcohol use.<br><br>As working conditions worsen, depression and *desesperación* increase.<br><br>As living conditions worsen, depression, *desesperación* and alcohol use increase.	Cross-sectional design limiting ability to establish direction of causality.<br><br>Generalizability of findings are limited by convenience sampling.

Citation	Purpose	Sample & Setting	Design & analytic strategy	Definition of Structural vulnerability	Operationalization of Structural Vulnerability	Health Outcomes/Related Factors	Key Findings	Limitations
Pérez-Figueroa et al. (2022)	To evaluate the association between key contextual factors and experiencing a non-fatal opioid overdose among people who use heroin.	101 people who use heroin<br><br>Median age = 40 years (range = 18 – 72)<br><br>73% Male<br><br>62.4% Latinx<br>11.9% Black<br>4.9% Multiracial<br><br>68.3% Equal to or lower than a high school education<br><br>100% reported heroin use in the last 3 months<br><br>53.5% experienced a non-fatal overdose<br>13.9% experience a non-fatal overdose in the past three months<br><br>April – June 2019<br><br>Washington Heights, New York	Cross-sectional, analytic<br><br>Data collected via surveys<br><br>Designed and implemented using principles of community based participatory research (CBPR)<br><br>Bivariate analyses<br>Multivariable logistic regression	Authors indicate "structural vulnerability exposes this population [study sample] to discrimination and social marginalization, which further increases overdose risk and constrains access to needed health care services and pursuit of healthier lifestyles."	Measures specifically incorporated to reflect structural vulnerability were not made clear.<br><br>The following were analyzed as "predictors of interest":<br>Age<br>Race<br>Not partnered<br>Hungry in last 6 months<br>Housing instability<br>Monthly income ≤ $800<br>Drug use	Non-fatal opioid overdose<br>Lifetime experience of overdose<br>Overdose in past 3 months<br><br>Measured as outcome/dependent variables	Key findings showed that among participants who had ever experienced a non-fatal opioid overdose, 68.5% were people of color and 31.5% were White.<br><br>Housing instability was common in this group (61%), with 41% experiencing homelessness—most of whom (78.6%) slept in places not meant for human habitation.<br><br>Non-fatal overdose was associated with race/ethnicity, being partnered, and injection drug use.<br><br>Additionally, younger participants who reported hunger in the past six months were more likely to have experienced a recent non-fatal overdose	Cross-sectional design limiting ability to establish direction of causality.<br><br>Majority of this sample had health insurance and prior exposure to drug treatment limiting the generalizability to people without insurance or prior drug treatment<br><br>Possible biases associated with the data collection strategy include social desirability, recall, and intoxication.<br><br>No power analysis reported to support selected sample size; Wide confidence intervals indicate imprecise statistical outcomes<br><br>Lack of clarity on which measures Authors theoretically aligned with structural vulnerability indicators

Citation	Purpose	Sample & Setting	Design & analytic strategy	Definition of Structural vulnerability	Operationalization of Structural Vulnerability	Health Outcomes/Related Factors	Key Findings	Limitations
Reilly et al. (2015)	To characterize indicators of structural vulnerability associated with HIV/STI risk behavior and explore the effect of accumulated vulnerability on the likelihood of dancers' engagement in risk behavior.	101 female exotic dancers (FEDs) working on "The Block, a historic red light district in downtown Baltimore."<br><br>Median age = 24 years<br><br>57.4% White<br>42.6% Non-white/other<br><br>July 2008 – February 2009<br><br>Baltimore, MD	Cross-sectional, analytic<br><br>Secondary data analysis from a study examining drug- and sex-related risk behaviors among FEDs<br><br>Data collected via surveys<br><br>Multivariate logistic regression	The impact of social and economic stressors experienced by female exotic dancer given their employment status and associated income	Structural vulnerability was measured using a cumulative indicator score based on four binary indicators:<br><br>Unstable housing living in boarding house, on the street, or in someone else's apartment, in past three months<br><br>Residential transience Moving >2 times in past year<br><br>Ever in jail<br><br>Illegal income sources<br><br>Each indicator was assigned a score of 0 or 1, producing a total vulnerability score ranging from 0 to 4.<br>Scores were then dichotomized into low vulnerability (0–1 indicators) and high vulnerability (2–4 indicators).<br>These vulnerability levels were used as predictors in regression analyses to assess associations with key health and behavioral outcomes.	HIV/STI risk<br>Drug use<br>Sex exchange<br>Multiple sex partners<br><br>Measured as binary outcome/dependent variables	Nearly half of participants (49%) reported unstable housing, 28% were transient, 55% had been jailed, and 30% reported earning income illegally.<br><br>HIV/STI risk behaviors were highly prevalent, with each reported by at least 40% of participants.<br><br>All structural vulnerability indicators were associated with recent drug use and sex exchange, while unstable housing and incarceration were also linked to multiple sex partnerships.<br><br>Increases in cumulative vulnerability scores were associated with higher frequency of drug use, sex exchange, and multiple partners, indicating a dose–response relationship between structural vulnerability and HIV/STI risk.	Cross-sectional design limiting ability to establish direction of causality.<br><br>The data are self-reported, social desirability bias may exist.<br><br>The small sample impacted the precision of the estimates for relationships under study.<br><br>A dichotomized, rather than continuous, vulnerability score was used which limited the variability of the outcome measure for the statistical analysis

Citation	Purpose	Sample & Setting	Design & analytic strategy	Definition of Structural vulnerability	Operationalization of Structural Vulnerability	Health Outcomes/Related Factors	Key Findings	Limitations
Schneider et al. (2024)	To examine differences in sleep context and characteristics and to assess associations of sociodemographic and substance use characteristics with sleep related impairments.	170 people who use opioids (PWUO)<br><br>Mean age 41.6<br><br>21.2% non-Hispanic White<br>66.7% non-Hispanic Black<br>12.1% Other race/ethnicity<br><br>November 2019 – March 2020<br><br>Anne Arundel County, MD	Cross-sectional, analytic<br><br>Secondary data analysis of survey data from the Peer harm Reduction of Maryland Outreach Tiered Evaluation (PROMOTE) study conducted in Baltimore and Arundel.<br><br>Regression analyses were conducted to examine associations between structural vulnerability, sleep-related outcomes, and other health behaviors.	Not specified	Structural vulnerability was operationalized through:<br>Housing instability<br>Food insecurity<br><br>These factors were incorporated as predictors in regression analyses to examine their relationships with sleep quality, impairment, and patterns.	Sleep health<br>Context<br>Quality<br>Schedule<br><br>PROMIS Sleep-Related Impairment measure	Sleep is a critical but often overlooked concern among PWUO. Over 25% of participants identified it as their most urgent need—above drug use or mental health.<br><br>Sleep-related impairment was widespread. Sleep disturbances were nearly universal across the sample and not limited to any single substance.<br><br>Mental health issues, particularly nightmares, stress, anxiety, and depression, were the most commonly reported contributors.<br><br>Structural vulnerabilities such as homelessness and hunger were the strongest predictors of poor sleep, even when controlling for substance use.<br><br>Authors linked these findings to the need for interventions that address both mental health and basic survival needs to improve sleep and overall well-being in this population.	Cross-sectional design limiting ability to establish direction of causality.<br><br>The data are self-reported, social desirability bias may exist.<br><br>No power analysis reported to indicate the adequacy of the sample size<br><br>Conceptualization of structural vulnerability is not clearly articulated

Citation	Purpose	Sample & Setting	Design & analytic strategy	Definition of Structural vulnerability	Operationalization of Structural Vulnerability	Health Outcomes/Related Factors	Key Findings	Limitations
Schneider et al. (2022)	To explore the longitudinal relationship between violence and drug use among a sample of women who exchange sex (WES) from Baltimore City, Maryland.<br>To examine how baseline indicators of socioeconomic and structural vulnerability are associated with drug use and experiences of violence.	251 street based cisgender women who exchange sex for goods<br><br>Mean age 37.8. year<br><br>57.4% non-Hispanic White<br><br>59.4% homelessness<br>58.6% weekly hunger<br><br>September 2017 – January 2019<br><br>Baltimore, MD	Longitudinal, two group comparison<br><br>Data collected via survey at baseline and at 6 months<br><br>Secondary analysis of data from the Enabling Mobilization, Empowerment, Risk Reduction, and Lasting Dignity (EMERALD) Study, a two-group comparison study evaluating a place-based empowerment intervention for women<br><br>Path analysis to assess the longitudinal effects of baseline drug use and separately for violence on drug use and violence at follow up.	Not specified	Structural vulnerability was operationalized using two binary indicators:<br>(1) homelessness in the past six months and<br>(2) experiencing hunger on a weekly basis in the past three months.<br><br>These indicators were included in the path model as predictors, with direct paths specified to baseline violence and drug use.	Experiences of violence by type and perpetrator<br><br>Physical violence<br>Sexual violence<br>Violence with a weapon<br><br>Non-paying sex partners<br>Paying clients<br>Other people<br><br>All combinations to form nine binary indicators; total score 0 - 9<br><br>Drug use<br>Seven specific drugs<br><br>Frequency of use (0 -3); total drug score – sum of frequency scores for all drugs (0-21)<br><br>Measured in path analysis: baseline violence and drug use with direct paths to violence and drug use at follow up.	Violence was common for the participants; the prevalence of violence types/perpetrator combinations ranged from 10.0% to 32.1%; and the prevalence of daily use of individual drugs was up to 70.8%.<br><br>Homelessness and hunger were significantly associated with increased violence and drug use, at baseline.<br><br>Hunger and homelessness were significantly associated with increased victimization and drug use scores at baseline; suggesting that violent victimization perpetuates drug use.	Race variable dichotomized to White and Non-White to ensure sufficient cell sizes<br><br>No power analysis reported to indicate the adequacy of the sample size<br><br>Conceptualization of structural vulnerability is not clearly articulated

Citation	Purpose	Sample & Setting	Design & analytic strategy	Definition of Structural vulnerability	Operationalization of Structural Vulnerability	Health Outcomes/Related Factors	Key Findings	Limitations
Sherman et al. (2024)	To examine the relationships between co-occurring stigma exposure, social and structural vulnerabilities, and mental health and identify common characteristics among transgender women to improve intervention tailoring for this group.	1418 participants assigned male at birth and identify as women or on a spectrum of feminine gender identity.<br><br>Two parallel cohorts:<br>892 In-person<br>526 Online-only<br><br>Mean age = 33 years (range 18 – 76)<br><br>53.8% People of color<br>46.2% Non-Hispanic White<br><br>Midwest 7.5%<br>Northeast 39.1%<br>South 0.2%<br>West 53%<br><br>March 2018 - August 2020<br><br>Various US regions<br>Midwest, Northeast, South, West	Cross-sectional, analytic<br><br>Data collected via surveys<br><br>Secondary data analysis of data from the Leading Innovation in Transgender Health and Empowerment (LITE), a prospective cohort study.<br><br>Guided by community advisory board for study conception, launch, implementation and dissemination<br><br>Partial least squares path modeling<br>Response Based Unit Segmentation (REBUS) algorithm to identify clusters<br>Multivariable linear regression	"Complex social and structural vulnerabilities refer to the co-occurrence of factors that decrease one's ability to recover or be resilient in response to stressful stimuli"	Structural vulnerability was operationalized using a range of binary, ordinal, and continuous variables, including:<br><br>Sex work<br>Lifetime; yes/no<br>Housing insecurity.<br>Never vs lifetime<br>Health insurance status<br>Private, public or uninsured<br>Employment<br>Full-time, part-time or unemployed<br>Food insecurity<br>Adapted from USDA Food Insecurity measures<br>Substance use<br>Drug Abuse Screening Test (DAST)<br>Social support<br>Medical Outcomes Study Social Support Survey<br><br>These variables were included as predictors in regression analyses to examine their associations with the outcomes of interest.	Mental health outcomes<br>PTSD<br>Psychological distress<br><br>Measured as an outcome/dependent variables in regression analysis<br><br>———<br><br>Stigma<br>Polyvictimization<br>Discrimination<br><br>Measured as a predictor in regression analysis	Results indicated that many transgender women face high risk for adverse mental health outcomes due to combined experiences of stigma and complex social and structural vulnerabilities<br><br>The analysis of the clusters demonstrated disparities in mental health severity<br><br>The PLS-PM models offered a "more complete model and better fit compared to the regression models."	Cross-sectional design limiting ability to establish direction of causality<br><br>Internal consistency of the instruments used to measure mental health outcomes not reported for the study sample.

Citation	Purpose	Sample & Setting	Design & analytic strategy	Definition of Structural vulnerability	Operationalization of Structural Vulnerability	Health Outcomes/Related Factors	Key Findings	Limitations
Sherman et al. (2019)	To better characterize the shared and distinct structural vulnerabilities of transgender female sex workers and cisgender female sex workers; and unpack how socio-structural factors synergistically and independently drive women's entry into sex work and shape the context in which HIV infection and risk-taking occur.	313 cisgender and transgender women involved in sex work<br><br>Total Sample<br>Mean age = 34<br>33.3% Black<br>13.5% Hispanic or other<br>53.2% White<br><br>Cisgender female sex workers (CFSW; n = 250)<br>Mean age = 36<br>22.8% Black<br>10.8% Hispanic or other<br>66.4% White<br><br>Transgender female sex workers (TFSW; n = 63)<br>Mean age = 30<br>75.8% Black<br>24.2% Hispanic or other<br>0% White<br><br>April 2016 - August 2017<br><br>Baltimore, MD	Cross-sectional, analytic<br><br>Data collected via surveys and biological specimens<br><br>Secondary data analysis of the Sex Workers and Police Promoting Health in Risky Environments (SAPPHIRE) study a prospective cohort study<br><br>Partnership with a community advisory board<br><br>Bivariate logistic regression<br>Multivariate logistic regression	"The positionality of an individual in a hierarchical social order."	Structural vulnerability was operationalized using multiple indicators measured as predictors in regression analysis, including:<br>Housing instability<br>Homelessness in the past 3 months<br><br>Financial instability<br>Legal unemployment and no monthly savings in the past 3 months<br><br>Food insecurity<br>Going to sleep hungry at least once a week<br><br>Limited education<br>Completion of high school<br><br>Criminal justice involvement<br>History of arrest or incarceration	HIV outcomes<br><br>Measured as an outcome/dependent variables in regression analysis<br><br>———<br><br>Substance use<br>Past 3 months<br><br>Exposure to client or police violence<br>Physical or sexual, ever<br><br>Measured for comparison of means between groups	Both CFSWs (cisgender female sex workers) and TFSWs (transgender female sex workers) reported economic hardship and lifetime exposure to client and police violence.<br><br>CFSWs faced significantly higher structural vulnerability, with greater rates of homelessness, food insecurity, lack of savings, low education, incarceration, and legal unemployment.<br><br>Food insecurity was associated with higher odds of HIV infection among CFSWs, and limited education, unemployment, and arrest history were linked to higher HIV risk among TFSWs.<br><br>Most TFSWs were Black, while the CFSW group was predominantly white.	Cross-sectional design limiting ability to establish direction of causality.<br><br>Limited generalizability due to the racial composition of the CFSW and TFSW groups.<br><br>There may have been additional drivers of HIV risk that were not captured in this analysis

Citation	Purpose	Sample & Setting	Design & analytic strategy	Definition of Structural vulnerability	Operationalization of Structural Vulnerability	Health Outcomes/Related Factors	Key Findings	Limitations
Tomko et al. (2023)	To study the relationship between co-occurring structural vulnerabilities and mental health in a population of female sex workers at high risk for HIV using latent class analysis.	385 cisgender female sex workers<br><br>Mean age = 37 years<br><br>57% White<br>46% had less than a high school education<br>26% of the sample, sex work was their only income source.<br><br>Women were recruited from area identified through geospatial analyses of potential sex worker locations<br><br>September 2017 – February 2019<br><br>Baltimore, MD	Cross-sectional, analytic<br><br>Data collected via surveys and biological specimens<br><br>Secondary analysis of baseline data from the Enabling Mobilization, Empowerment, Risk Reduction, and Lasting Dignity (EMERALD) Study, a two-group comparison study evaluating a place-based empowerment intervention for women<br><br>Descriptive statistics<br>Latent class analysis (LCA)	Structural vulnerability exists "when an individual or group's position in society constrains behavior due to conflict with" existing hierarchies defined and ordered by perceived "worthiness;" historically defined norms and ethics; and the medicalization of individual characteristics or life circumstances" such as homelessness, "that can produce social exclusion and constrain opportunities for optimal health and security."	Structural vulnerability was operationalized using binary (yes/no) indicators measured over the past six months, including:<br><br>Housing security<br>Living in more than two locations<br><br>Food insecurity<br>Going to sleep at night hungry; not enough food at least once per week<br><br>Financial dependence on someone else<br><br>Client-perpetrated physical violence<br><br>Client-perpetrated sexual violence<br><br>These indicators were incorporated as latent class indicators in the analysis.	HIV risk<br>Injection drug use<br>Condomless sex<br>STI infection<br><br>Mental health<br>Depression<br>PTSD<br>Mental distress<br><br>Measured as binary outcome/dependent variables	High levels of structural vulnerability were reported in the sample, with 74.3% experiencing housing insecurity, 47.8% food insecurity, 48.8% financial dependence, 31.9% physical violence, and 25.8% sexual violence.<br><br>Latent class analysis identified three groups: Minimal Structural Vulnerability (MSV, 43%), Material Needs (MN, 29%), and High Structural Vulnerability (HSV, 28%).<br><br>The MN class had high housing and food insecurity but low violence exposure, while HSV had high probabilities across most indicators.<br><br>Compared to MSV, both MN and HSV showed higher rates of injection drug use and greater mental health burden (depression, PTSD, and distress).<br><br>HSV also reported higher levels of condomless sex compared to both MSV and MN.	Cross-sectional design limiting ability to establish direction of causality.<br><br>Data derived from self-report is subject to recall and social desirability biases

Citation	Purpose	Sample & Setting	Design & analytic strategy	Definition of Structural vulnerability	Operationalization of Structural Vulnerability	Health Outcomes/Related Factors	Key Findings	Limitations
Urquhart et al. (2021)	To characterize associations of individual, interpersonal, and structural factors with frequent restless sleep among female sex workers.	236 cisgender female sex workers<br><br>Median age = 35 years<br><br>68% White<br>22% Black<br>10.2% Hispanic/Other<br><br>April 2016 - January 2017<br><br>Baltimore, MD	Cross-sectional, analytic<br><br>Data collected via survey<br><br>Secondary data analysis of the Sex Workers and Police Promoting Health in Risky Environments (SAPPHIRE) study a prospective cohort study<br><br>Partnership with a community advisory board<br><br>Descriptive statistics<br>Pearson's X^2<br>Multivariable logistic regression<br><br>Pearson's X^2 tests were used to assess differences by restless sleep frequency; variables significant at the $P < .05$ level were considered for model inclusion	"Constrained resource access due to one's position within social, economic, and political structures, such as people who use drugs, police- and violence-exposed populations and those who experience food and housing insecurity."	Structural vulnerability was operationalized using binary indicators, including:<br>Education<br>non-high school graduate vs. higher<br><br>Food insecurity<br>recently went to bed hungry 1+ night per week vs. 0<br><br>Housing insecurity<br>recently lived in 3+ places vs. fewer<br><br>Recent homelessness<br>Yes vs No<br><br>Food insecurity included as a predictor in regression analysis	Sleep<br>Number of days with restless sleep in the past week.<br>Frequent: 5-7 days<br>Less frequent: 0-4 days<br><br>Measured as a binary outcome/dependent variable in regression analysis<br><br>---<br><br>Substance use<br>Self-rated General health<br><br>PTSD symptoms<br>PCL-5<br><br>Cumulative violence exposure<br>The sum of each type of lifetime violence<br><br>Self-rated health and cumulative violence included as predictors in regression analyses.	52% lacked a high school diploma<br>61% weekly food insecurity<br>55% recently housing insecure<br>62% recently homeless<br><br>FSW experience high levels of sleep disturbance and concurrent vulnerabilities which may amplify risk health effects of poor sleep, such as insomnia.<br><br>Age≥ 35 years, self-rated poor health, and weekly food insecurity were independently associated with frequent restless sleep	Cross-sectional design limiting ability to establish direction of causality.<br><br>Number of traumas experienced reported as statistically significant finding; measure is not defined or described.

Mixed Methods Studies

Citation	Purpose	Sample & Setting	Design & analytic strategy	Definition of Structural vulnerability	Operationalization of Structural Vulnerability	Health Outcomes/Related Factors	Key Findings	Limitations
Friedman et al. (2019)	To characterize the structural vulnerability of the Puerto Rican community in Philadelphia to gun violence using a mixed methods approach, and to present an assessment of firearm violence in these communities that is quantitatively rigorous while conveying the lived experience of the human suffering reflecting in the macro statistics.	Sample included an unspecified number, "dozens of respondents"<br><br>Sample sociodemographic information unspecified<br><br>"Philadelphia's sprawling open-air narcotics market located in the heart of the city's Puerto Rican area."<br><br>2006 – 2017<br><br>Philadelphia, PA	Exploratory sequential mixed methods<br><br>Data collected via intensive participant-observation, conversational interviews and immersion in the social environment over the course of 6 years.<br><br>Qualitative analysis of ethnographic data to generate hypotheses about macro-level dynamics for quantitative testing<br><br>Quantitative analysis of geo-referenced data using Poisson regression<br><br>Iterative comparison of the qualitative and quantitative results.	" Structural vulnerability is driven by power relations- large scale social and economic forces that marginalize certain individuals; The concept engenders an understanding of how local cultural and social dynamics often mediate or exacerbate personal risk of illness."	Authors indicate that participants were "rendered vulnerable to violence by structural factors such as their low educational levels, marginalization from legal employment opportunities, easy access to wholesale supplies of heroin and cocaine, easy access to licensed and unlicensed firearms, and alienation from social services."	Cumulative exposure to violence	Poverty was significantly associated with violence for all social groups; Puerto Rican neighborhoods were disproportionately impacted.<br><br>Per capita murder rate of the field site was 62 per 100,000 persons per year, 10x higher than majority white areas, and 3x higher than the city average<br><br>There were virtually no legal businesses offering meaningful employment opportunities<br><br>The narcotics economy, an "equal opportunity" employer, operates with a hierarchy of roles and as a self-contained instance of exploitative capitalism – the lowest level employee experiences the most risk.<br><br>Gun violence is ubiquitous and routinely used to maintain power, protect resources, and maintain order.<br><br>Participants were rendered "structurally vulnerable" to violence due to social and economic forces that perpetuate high levels of violence in poor non-white communities.	Data source - police data - present a risk of bias given the under-counting of the prevalence of violence and narcotics.<br><br>Quantitative analysis is ecological – authors describe neighborhood level associations at the census tract level.

Citation	Purpose	Sample & Setting	Design & analytic strategy	Definition of Structural vulnerability	Operationalization of Structural Vulnerability	Health Outcomes/Related Factors	Key Findings	Limitations
Friedman et al. (2021)	To assess how experiences with abusive or violent policing among people who inject drugs relate to intersectional structural vulnerability.	494 people who inject drugs (PWID) in the survey sample;<br>60.5% Male<br>Mean age = 44.2 years<br>59% people of color<br><br>54 PWID in the qualitative sample;<br>8 key informants<br>46 PWID<br>Demographic composition not specified beyond "relatively similar" to the quantitative sample.<br>Mean age = 38.7 years<br>65% Male<br>37% Hispanic<br><br>"Harm reduction spaces," churches and health clinics.<br><br>March – December 2015<br><br>Fresno and Kern Counties, California	Exploratory, sequential, mixed methods<br><br>Data collected via ethnographic field work, interviews, and surveys<br><br>Bivariate and multivariate regression analysis<br>Exploratory, descriptive data visualization to identify clusters of intersectional vulnerability.	"The framework of structural vulnerability highlights how each individual's risk of a deleterious health outcome—such as experiencing police brutality—is related to their position in the wider social and economic hierarchies of their local environment."	Structural vulnerability (SV) is operationalized as a theoretical framework with intersectionality theory, forming *intersectional structural vulnerability* – an approach to exploring how "multiple overlapping and interrelated demographic, personal identity, occupational and social characteristics interface in nonlinear ways."<br><br>For quantitative analysis, SV operationalized as:<br>"Vulnerability factors" (independent variables)<br>Gender<br>Race<br>Level of educational attainment<br>Current housing status<br>Currently residing in a rural location<br>Having ever engaged in sex work.	Abusive or violent policing<br>Physical assault<br>Verbal abuse<br>Sexual violence or exploitation<br>Confiscation of unused syringes<br>Number of times stopped but not arrested	Police harassment impacted participants' everyday lives manifesting as routine searches at their places of work or in their homes.<br><br>Police violence routinely occurred and was incited by an officer's perception of a threat to their authority or safety.<br><br>There was an overall positive relationship between the number of vulnerability factors and the average police interaction score<br><br>Frequency of abusive encounters with police:<br>Physical violence (42%)<br>Verbal abuse (62%)<br>Sexual violence (9%)<br>Unwarranted confiscation of unused syringes (39%)<br><br>Females reported higher rates of sexual violence; males reported higher rates of physical violence.<br><br>Rates of police-perpetrated sexual violence and exploitation varied	Sample size placed limitations on the methodologies employed – interaction terms in regression analysis were not viable.<br><br>Race measured as a binary variable, limiting possibilities for statistical analysis.

Qualitative Studies

Citation	Purpose	Sample & Setting	Design & analytic strategy	Definition of Structural vulnerability	Operationalization of Structural Vulnerability	Health Outcomes/Related Factors	Key Findings	Limitations
Arnold et al. (2021)	To describe the broad experience of personal and observed workplace injuries of Latinx child farmworkers in North Carolina as reported by the children themselves.<br><br>To highlight the ways in which Latinx children's labor in one of the most hazardous industries is produced and sustained by structural factors that increase their vulnerability constrain their agency, normalize preventable bodily harm, and largely conceal this reality from the public.	30 child farmworkers in thirteen different North Carolina counties.<br><br>Age range 10 – 17 years<br>10 – 13 years (n = 9)<br>14 – 17 years (n = 21)<br><br>13 girls<br>7 boys<br><br>15 were born in the US<br>11 were born in Mexico<br>4 were born in Guatemala<br><br>June – September 2016<br><br>North Carolina	In-depth interviews<br><br>Community-based participatory research approach<br><br>Data analysis occurred utilizing a summary matrix to classify the data with "occupational injury" and "work risk" codes.	"The ways in which various form of exploitation, racialized discrimination, and symbolic subjectivities combine to create a positionality in which groups are subjected to forms of violence in patterned ways."	Structural vulnerability operationalized as a theoretical lens to analyze the context of Latinx child agricultural worker injury	Occupational injury<br>Risk of bodily injury<br>Farmwork related illnesses	Most participants described experiences of injury ranging in severity from minor scratches to blunt trauma and green tobacco sickness<br><br>The agricultural work experience is a persistent "risk environment" that is normalized; Participants' responses generated the theme, "get used to" it, indicating that time will allow for their acclimation to exposure to hazards<br><br>Work involvement as a minor is allowable by U. S. law. Seeking supplemental income to support the family was the driver of the child workers involvement in agricultural work<br><br>A lack of regulatory oversight exacerbated the lack of safety and substandard agricultural working conditions is the 'norm' in North Carolina.<br><br>Retained agency was constrained by structural factors. Participants risked losing their income if they prioritized their own safety.	The findings may not reflect the experience of Latinx child farmworkers in other states or of those working other crops.<br><br>Some participants began working at the age of six, increasing the risk of participants' recall bias

Citation	Purpose	Sample & Setting	Design & analytic strategy	Definition of Structural vulnerability	Operationalization of Structural Vulnerability	Health Outcomes/Related Factors	Key Findings	Limitations
Arnold et al. (2024)	To describe and contextualize Latine youth farmworkers experiences of work safety during the first 2 years of the COVID-19 pandemic by drawing from interviews with education, health, and non-profit advocacy service providers and Latine youth farmworkers	24 Latine youth farmworkers (migrating or seasonal)<br><br>Age range 10 – 13<br>12 girls<br>12 boys<br><br>10 service providers (providing services to farmworker families)<br><br>Age range 25 – 59<br><br>March – November 2022<br><br>North Carolina	In-depth interviews 2 phases<br><br>Community-based participatory research approach<br><br>Thematic analysis	"Due to macro-level social processes, [people] embody a social location in which exploitation, discrimination, and negative health outcomes are common"	Structural vulnerability operationalized as a theoretical lens to analyze the context of Latine youth experiences working in agriculture in North Carolina	Occupational injury	Latinx youth farmworkers experienced increased labor during the first two years of the COVID-19 pandemic.<br><br>Both youth and service providers reported that many felt they had no choice but to work, despite known risks of COVID-19.<br><br>Youth described unsafe working conditions, including inadequate protections and supervisors who prioritized productivity over safety.<br><br>Many also worked under physically strenuous conditions, such as extreme heat, and were potentially exposed to hazards like pesticides.<br><br>Some youth described their employers as "nice" or "generous," these positive impressions often referred to basic legal requirements, like providing water or rest breaks.<br><br>Some families even viewed their children's ability to work as a necessary or positive survival strategy, despite the risks involved.	The findings may not reflect the experience of Latine child farmworkers in other states.<br><br>Possibility of recall bias

Citation	Purpose	Sample & Setting	Design & analytic strategy	Definition of Structural vulnerability	Operationalization of Structural Vulnerability	Health Outcomes/Related Factors	Key Findings	Limitations
Brantley et al. (2017a)	To uncover how structural vulnerability is experienced at the individual level and to examine the interplay of structural drivers before and after initial entry into the exotic dance club environment.	24 exotic dancers working in Baltimore City and County exotic dance clubs<br><br>Median age = 21 years (range 19 – 33)<br><br>50% Black<br>33% White<br>17% Other ethnicities/races<br><br>n=24 Initial interviews<br>n=21 Second interviews<br><br>July 2014 – May 2015<br><br>Baltimore City and County, Maryland	Longitudinal, cohort<br><br>Semi-structured, in-depth interviews at two time-points (baseline and follow-up at 3 – 6 months)<br><br>Thematic content analysis	"The location or position in society where a concentration of multiple discriminations constrains individual agency for sexual-decision making."	Structural vulnerability operationalized as an analytic lens<br><br>The authors approach involved deductive development of codes related to theoretically informed social and economic aspects of structural vulnerability included in the interview guides.<br><br>An emphasis was placed on the dancers' social and economic circumstances to identify the overlapping themes to characterize the most prominent structural factors in which their vulnerability was rooted.<br><br>Dancers' perspectives on drug use and selling sex were extracted to examine the interplay between structural vulnerability and engaging in drug- and sex-related activity occurring in EDCs.	HIV/STI risk	Chronic scarcity of resources, transient living situations, childhood/youth adversity, and early independence propelled them into exotic dance work.<br><br>Housing instability, financial insecurity, criminal history and limited education and job opportunities were reasons for the participants remaining in the exotic dance club environment.<br><br>Accumulated vulnerability further constrained their opportunities to achieve economic and social stability<br><br>Higher vulnerability led to work related sexual risk behavior. Women who were able to improve their circumstances engaged in less sexual risk behaviors and maintained a greater degree of agency<br><br>Dancers described converging challenges. Accessing safe housing, affordable education, reliable employment and legal support were noted as persistent challenges.<br><br>Dancers with social networks and familial support were able to mitigate the effects of structural vulnerability.	Risk of recall and social desirability bias due to questions about private or illegal behaviors

Citation	Purpose	Sample & Setting	Design & analytic strategy	Definition of Structural vulnerability	Operationalization of Structural Vulnerability	Health Outcomes/Related Factors	Key Findings	Limitations
Chang et al. (2019)	To examine the narratives of people who inject drugs surrounding their recent overdose experiences.	40 people who inject drugs (PWID)<br><br>72% male<br><br>63% White<br>15% Black<br>20% Hispanic<br>3% Multiracial<br><br>Mean age = 43 years<br><br>88% had experience with substance use treatment<br>100% experienced an opioid overdose within 5 years<br>52% overdosed at least once in the past 12 months.<br>72% witnessed at least one overdose in the past 12 months<br><br>Recruited from syringe service programs<br><br>2014 – 2015<br><br>San Francisco, California	Semi-structured interviews<br><br>Content analysis	"The human-made political, economic, and social organization of risk environments." The "power and powerlessness contingent on one's social position" - often defined by race, class, gender, sexuality, and other categories – that shapes health risks in patterned ways.	Structural vulnerability operationalized as a theoretical framework for examining the violence that is structured into the lives of PWID, with recognition that there is a dynamic relationship between PWID and the risk environments they inhabit.<br><br>SV operationalized alongside the theory of lay expertise to situate and interpret the participants narratives	Opioid overdose	Illness, death, separation and persistent stress resulting from participants' structural vulnerability shaped the overdose narratives<br><br>Participants' express their opioid expertise - in-depth knowledge about opioid risks - in the context of survival within a risky environment.<br><br>PWID who practiced harm reduction are vulnerable to the risk of opioid overdose due to the rise in synthetic Fentanyl coupled with a lack of resources to ascertain the purity of the heroin.<br><br>Participants linked their drug use in response to emotional pain to the structural vulnerability they faced.<br>*"When I was depressed…I had to use in order to just try to stay high and forget."*	Sampling strategy – at a syringe service program (SSP) – increased the likelihood that the findings do not represent the perspectives of people who do not have access to an SSP.

Citation	Purpose	Sample & Setting	Design & analytic strategy	Definition of Structural vulnerability	Operationalization of Structural Vulnerability	Health Outcomes/Related Factors	Key Findings	Limitations
Collins et al. (2024)	To understand the alcohol-related treatment and support needs of unstably housed individuals with high-intensity alcohol use in Rhode Island.<br><br>The study explores how structural barriers, stigma, and the lack of accessible, non-abstinence-based programs shape vulnerability to alcohol-related harms.<br><br>The goal is to inform more inclusive, harm-reduction-oriented services that account for the lived realities and treatment preferences of people experiencing both housing instability and heavy alcohol use.	25 unstably housed individuals who self reported heavy alcohol use.<br><br>Mean age 41.7<br><br>20% Black<br>52% White<br>8% Hispanic/Latino<br>20% More than one race<br><br>32% Women<br>68% Men<br><br>100% Cisgender<br><br>Rhode Island<br><br>June 2021 – July 2022	Semi-structured interviews and participant observation<br><br>Thematic analysis	"The concept of structural vulnerability draws attention to how social suffering is produced and reproduced through intersecting impacts of social-structural forces at play within social hierarchies that render certain people more susceptible to harm."	Structural Vulnerability operationalized as a Framework supporting the analysis focused on how structural inequities—such as poverty, unstable housing, exclusion from healthcare systems, and cyclical engagement with abstinence-based treatment programs—shaped participants' lived realities.	Alcohol use<br>Alcohol treatment<br>Support needs	Housing instability was found to be a primary driver of high-intensity alcohol use among unstably housed individuals, with alcohol often used to cope with stress and insecurity.<br><br>While participants frequently engaged with treatment programs, most found them ineffective due to rigid, abstinence-focused models that failed to meet their needs—especially for those not aiming for sobriety or managing polysubstance use.<br><br>The findings emphasize the need for a broader continuum of care, including harm reduction approaches like Managed Alcohol Programs, low-barrier housing, and drop-in consumption spaces.	All participants were cisgender, which limits the understanding of experiences among non-binary, transgender, genderqueer, or gender non-conforming individuals.<br><br>Using national guidelines for heavy drinking may have excluded individuals with extremely high-intensity alcohol use or differing patterns, potentially limiting the range of treatment needs captured.<br><br>The findings are based on programs and participants in one U.S. state (Rhode Island), limiting generalizability to other regions.<br><br>Data were collected at one point during the COVID-19 pandemic, limiting the ability to assess changes over time or long-term engagement with service.

Citation	Purpose	Sample & Setting	Design & analytic strategy	Definition of Structural vulnerability	Operationalization of Structural Vulnerability	Health Outcomes/Related Factors	Key Findings	Limitations
Haas et al. (2018)	Utilize a structural vulnerability framework to more broadly define the hazards influencing high work-related musculoskeletal disorders (WMSD) rates among Certified Nursing Assistants (CNAs); and to demonstrate the utility of structural vulnerability theory in occupational safety and health (OSH) research more broadly.	26 certified nursing assistants (CNAs) working in Nursing and Residential Care Facilities<br><br>Mean age = 45 years (range = 20 to 63)<br><br>96% (n = 25) Female<br><br>73% White<br><br>55% reported income less than $35,000/year<br><br>Back and shoulder injuries were the most common<br><br>Washington state<br><br>2011 - 2014	Qualitative interviews<br><br>Exploratory, qualitative analysis	"The individual experience of the forces of structural violence."	Structural vulnerability operationalized as an analytic framework to broadly define the hazards influencing rates of injury among CNAs	Occupational injury<br>Work related musculoskeletal disorders	CNAs' descriptions of their WMSDs paint a picture of a work environment situated in a broader structural environment that appears to produce barriers to worker health and safety.<br><br>CNAs are situated at the bottom of a hierarchical system that affords them the lowest pay, and little power to control their health outcomes and injury risk.<br><br>Policies and practices that increase risk result from management priorities being diverted away from worker safety.<br><br>CNAs' experience compounded vulnerability characterized by low income, financial desperation, working multiple jobs to make ends meet, and relying on welfare when experiencing an injury-related pay reduction. With limited resources both at work and at home, CNAs experience a "double-whammy" of vulnerability.	Specific interview questions regarding structural factors were absent, and the study was conducted with an under-sampling of racially and ethnically minoritized groups.

Citation	Purpose	Sample & Setting	Design & analytic strategy	Definition of Structural vulnerability	Operationalization of Structural Vulnerability	Health Outcomes/Related Factors	Key Findings	Limitations
Holmes (2011)	To analyze hierarchies of ethnicity and citizenship, structural vulnerability, and health disparities in agriculture in the United States	Unspecified number of Participants<br><br>Farm labor hierarchy:<br>10 Farm executives<br>3 Crop managers<br>10 Supervisors<br>10 Administrative assistants<br>20 Checkers<br>60 Hourly field workers<br>300 Contract field workers<br><br>The Tanaka Farm<br><br>Skagit County, Washington<br><br>Year not specified	Ethnography<br><br>Data collected via participant observation over 1 year<br><br>Presented as “thick descriptions” of the complex hierarchy	Structural vulnerability “trains the gaze onto the social structures that produce and organize suffering into what public health denotes as health disparities,” directing “blame and interventional attention away from individuals and toward the social structures producing” their vulnerability.	A seminal work that explored structural vulnerability as a consequence of the social and structural hierarchies	Occupational injury Symptoms and pain related to the manual labor of picking	Powerful inputs into the suffering of farm workers were structural, and not willed by individual agents.<br><br>Individuals (white adolescents and young adults) learned and reinforced a position of power over Mexicans workers, including those “old enough to be their parents or grandparents.”<br><br>The “Continuum of Structural Vulnerability” is evident as “everyone on the farm is structurally vulnerable, although the characteristics and depth of vulnerability change depending on one’s position within the labor structure.“<br><br>“Marginalization begets marginalization,” structural vulnerability is reinforced by official and unofficial policies that prescribe social position, and define access to opportunities.<br><br>Race, citizenship and modernity were conflated as markers for access to employment opportunities	Limited discussion or descriptions of the occupational injuries and health conditions

Citation	Purpose	Sample & Setting	Design & analytic strategy	Definition of Structural vulnerability	Operationalization of Structural Vulnerability	Health Outcomes/Related Factors	Key Findings	Limitations
Kohut et al. (2024)	This study sought to understand and reduce the barriers to COVID-19 testing faced by structurally vulnerable populations in Portland, Maine, with a focus on individuals experiencing homelessness, immigrants, and those who are low-income or uninsured.<br><br>Specifically, the study aims were: to uncover the distinct obstacles these groups encounter when trying to access testing, and to explore how broader structural vulnerabilities—such as socioeconomic disadvantage and systemic inequities—shape and exacerbate these challenges.	34 members of structurally vulnerable populations<br><br>People experiencing homelessness<br>Immigrants, refugees, and asylum seekers<br>People who inject drugs (PWID)<br>Other individuals accessing public services (e.g., STD Clinic, Free Clinic, Needle Exchange)<br><br>27 key informants<br><br>Portland, Maine<br><br>October 2021 – April 2022	Semi-structured interviews<br><br>Thematic analysis	"Structural vulnerabilities encompass the socioeconomic, political, and cultural power hierarchies/systems that hinder the ability for certain groups to engage in healthcare and, consequently, put them at greater risk for poorer health outcomes."	Structural vulnerability was operationalized as an analytic lens through which the research team examined participants' testing experiences. Specific structural conditions—such as poverty, housing instability, and immigration status—were identified from participants' narratives. These conditions were then used to reanalyze coded data to understand how they exacerbated barriers to COVID-19 testing.	COVID-19 testing	Structural vulnerabilities—such as economic and legal precarity, language barriers, a confusing healthcare system, and stigmatizing medical experiences—significantly undermined access to and willingness to seek COVID-19 testing among structurally vulnerable populations.<br><br>These conditions created tangible risks for individuals, including financial strain from missing work, legal concerns about surveillance, and psychological harm from engaging with mistrustful or stigmatizing systems.<br><br>Together, these barriers not only disincentivized testing but also deepened inequities in access to medical care.	Limited sampling for certain immigrant groups (specifically Latino, Somali, and Arabic), due to recruitment challenges.<br><br>Non-representativeness of the sample. Participants do not reflect the full diversity or views of their broader communities.<br><br>Selection bias. Many participants were already engaged with health services, which may not capture the perspectives of those not accessing such services.<br><br>Restricted generalizability. The study avoids making broad conclusions about any specific group and instead focuses on summarizing the range of experiences observed

Citation	Purpose	Sample & Setting	Design & analytic strategy	Definition of Structural vulnerability	Operationalization of Structural Vulnerability	Health Outcomes/Related Factors	Key Findings	Limitations
Manser et al. (2024)	To explore barriers and facilitators to diabetes medication adherence and self-management among people with type 2 diabetes who have experienced homelessness.	26 participants with type 2 diabetes and lived experience of homelessness<br><br>Age range in years<br>23% 31 – 50<br>69% 51 – 70<br>8% 71+<br><br>42% Female<br><br>62% Black/African American<br>15% White<br>15% American Indian<br>20% Asian, Native Hawaiian, Pacific Islander, or Other<br><br>15% Hispanic or Latino<br><br>Housing at time of interview<br>35% Shelters<br>38% Supportive housing<br>19% No steady place/in car<br>7% Living with friends/family<br><br>Minneapolis, MN<br><br>October 2019 – March 2020	Exploratory, qualitative, cross-sectional study<br><br>One-time participant focus groups and individual in-depth interviews.<br><br>Analysis followed grounded theory principles with thematic analysis	Positionality within intersecting social, economic, and political hierarchies that impair capacity to engage in diabetes care. Includes factors like income, race, housing instability, and systemic discrimination.	Structural vulnerability was used as a lens to interpret how broader systemic forces shaped individuals' diabetes management challenges—e.g., unstable housing, stigma in shelters, disrupted care, and economic constraints.	Diabetes medication adherence<br>Self-management (e.g., glucose monitoring, diet)<br>Use of insulin and diabetes supplies<br>Barriers such as food insecurity, housing instability, access to care, and psychosocial distress.	Three interrelated themes:<br>Personal autonomy and security was impacted by unsafe environments, food insecurity, and theft of supplies.<br><br>Predictability and stability was challenged by transience, weather, and inconsistent routines.<br><br>Supportive, knowledgeable relationships were essential but often disrupted, including healthcare provider continuity and social support.<br><br>Also noted was a "domino effect" where loss of housing or support cascaded into worsening health.	Limited recruitment diversity due to COVID-19 and reliance on trusted intermediaries.<br>Participants may differ from harder-to-reach subgroups, like those unsheltered long-term or with severe mental illness.

Citation	Purpose	Sample & Setting	Design & analytic strategy	Definition of Structural vulnerability	Operationalization of Structural Vulnerability	Health Outcomes/Related Factors	Key Findings	Limitations
McKenna (2014)	To explore how women who use methamphetamine navigate sexual relationships, survival strategies, and drug acquisition in the context of structural vulnerability and risk environments, particularly focusing on exchange sex and reciprocity.	Over 30 individuals who use methamphetamine<br><br>25 identified as women<br><br>The women lived in economically precarious circumstances and most faced housing insecurity.<br><br>Denver, Colorado	Ethnography using participant observation, informal and group interviews, and direct/indirect questioning.<br><br>Analysis centered on women's experiences and broader sociocultural and political-economic contexts that shape risk and survival strategies.	Defined as the result of structurally rooted, gendered power inequities and institutional arrangements that shape individuals' exposure to harm, especially for women dependent on men for survival, material support, or drug access.	Used as an analytic lens to examine how gendered economic dependencies, housing insecurity, and criminalization shaped women's drug use and sexual relationships. Structural vulnerability is illustrated through women's limited agency in managing risk and securing resources.	Risk for HIV and STIs, violence, stigma, drug use behaviors (meth use and poly-drug use), access to housing and employment, and criminalization.	Most women did not engage in direct transactional sex but operated within relationships shaped by mutual expectations of reciprocity.<br><br>Meth use and relationships often intertwined with economic survival strategies.<br><br>Women's sexual practices and drug use were shaped by gendered power imbalances and structural conditions.<br><br>Risk and harm were embedded in social and economic relationships rather than solely individual behavior.	The findings are based on preliminary data and only women's perspectives are discussed, potentially limiting broader applicability. The article does not discuss male perspectives or generalize across all women who use meth.

Citation	Purpose	Sample & Setting	Design & analytic strategy	Definition of Structural vulnerability	Operationalization of Structural Vulnerability	Health Outcomes/Related Factors	Key Findings	Limitations
Organista et al. (2013)	To explore the sexual health and well-being of Latino migrant day laborers with attention to conditions of structural vulnerability.	51 Latino migrant day laborers (LMDLs)<br><br>Mean age = 38 years<br>33% between 21 - 30<br><br>Primarily from Mexico; secondarily from Central America<br><br>60% in the US less than 10 years<br><br>San Francisco and Berkeley, California<br><br>Year unspecified	Ethnography<br><br>Data collected over one year via naturalistic and participant observations and in-depth semi-structured interviews.<br><br>Part of a larger National Institute on Alcohol Abuse and Alcoholism research project to develop and test a structural-environmental model of alcohol-related sexual HIV risk and prevention among LMDLs.<br><br>Grounded theory approach	"Structural vulnerability describes the positionality of a population in society existing within harsh living and working conditions produced and reproduced by particular sets of global, economic, political, social and cultural factors."	A model of structural vulnerability allowed for conceptualizing HIV risk and sexual risk as a causal pathway stemming from distal structural and environmental factors considered "conditions of structural vulnerability":<br>Access to work<br>Living environments<br>Work environment	Sexual health and well-being	Three themes:<br>Familism and masculinity as the cultural context of sexual health<br>Frustration with limited options to meet life goals<br>The challenge of coping with threats to sexual health in the United States.<br><br>Participants shared that their goals to make economic progress and provide for a family were undermined by their structural vulnerability which manifested as prolonged searching for employment, reinforcing their need for day labor<br><br>Participants expressed goal frustration which resulted in 'falling into vices' including sexual risk taking and substance use.<br><br>Limited relationship options function as a precursor to sexual health choices. Their relationship options were contingent on the participants' social positionality.	Author noted – trade off between sample size and the generalizability, to broader population attributed to qualitative research in general.<br><br>Discussion on the living and working conditions was limited.<br><br>An integrated grounded theory generated from the analysis of the data is not made explicit

Citation	Purpose	Sample & Setting	Design & analytic strategy	Definition of Structural vulnerability	Operationalization of Structural Vulnerability	Health Outcomes/Related Factors	Key Findings	Limitations
Tulimiero et al. (2021)	To explore community health priorities and barriers to Latino immigrants' health care services use in rural communities, and participants' ideal model for health care services delivery	Unspecified number of Latino immigrants participated in in-home meetings<br>15 Latino immigrants who access care at a mobile clinic – participants in interviews<br><br>Participants identified as either Mexican or born in Mexico.<br><br>Over a third were women.<br><br>A majority identified Spanish as their primary language; other participants spoke an indigenous language<br><br>A majority had no health insurance<br><br>Eastern Coachella Valley, Southern California<br><br>Fall 2017 to Spring 2019	Semi-structured interviews and Focus groups<br><br>Community-based participatory research (CBPR) approach<br><br>Study carried out in two phases -<br>Phase I : Data collection<br>Phase-II: Intervention design, implementation and Evaluation<br><br>Guided by a community advisory board<br><br>Data analysis with template and matrix to identify patterns and emergent themes	"Structural vulnerability is an individual's or group's condition of being at risk for negative health outcomes through their interface with socioeconomic, political, and cultural/normative hierarchies."<br><br>"The concept of structural vulnerability shifts the focus from individual behaviors to the systems and power hierarchies that produce suffering and restrict the abilities of certain groups to control their circumstances and pursue healthy lifestyles."	Applied as a framework to understand the health inequity of Latino farmworkers as it is affected by systems and hierarchies.	Health care access	Participants descriptions of barriers to health services, included fear of deportation, distance to travel, cost of services relative to income, clinic hours, scheduling and wait times.<br><br>Participants described their healthcare access needs in the context of their structural vulnerabilities, suggesting hours and locations that would increase accessibility and use, and decrease their risk of deportation.<br><br>Design and implementation of the mobile clinics resulted in accessible care and participants descriptions included: "It's good that they [the medical students] see the necessities that there are in the community" and "It's good for them and also for us."	Social desirability bias may have impacted the study participants' account of the effectiveness of the student-run clinic.

Citation	Purpose	Sample & Setting	Design & analytic strategy	Definition of Structural vulnerability	Operationalization of Structural Vulnerability	Health Outcomes/Related Factors	Key Findings	Limitations
Westbrook (2024)	To examine how exclusionary displacement pressure shapes low-income residents' health and wellbeing over time.	35 residents in a predominantly low-income Hispanic/Latinx immigrant neighborhood<br><br>Mean age = 46 years<br><br>26 women<br>9 men<br><br>86% identified as Hispanic/Latinx<br><br>Over two thirds were undocumented<br><br>Denver, Colorado<br><br>2019-2022	Ethnography<br><br>Data collected through fieldwork and interviews over the course of 2.5 years<br><br>Thematic analysis	"Structural vulnerability is the risk of experiencing structural violence … and a product of economic exploitation based on discrimination by class, gender, sexuality, and race/ethnicity."<br><br>"Structural vulnerability theory reveals how uncontrolled housing cost increases, few rental support resources, and inadequacies in the social safety net lead politically marginalized groups toward poor health outcomes	Utilized as a theoretical framework along with Krieger's Ecosocial theory;<br><br>Guided by structural vulnerability, the author focused on the participants' lack of documentation status, inadequate work, and limited access to safety net systems when identifying how exclusionary displacement pressure is internalized and responded to as an embodied health experience.	Mental health<br>General well-being	Participants' experiences of exclusionary displacement pressure were detailed within three categories of Embodied Health Experiences: *Feeling, Battling and Enduring*<br><br>Feeling the pressure: Residents experienced stress, high blood pressure and depression due to displacement pressure.<br><br>Battling and struggling against the pressure: Residents embodied feelings of uncertainty, and limited control of their housing options, which manifested as stomachaches, headaches, and perseveration.<br><br>Enduring and bearing the pressure: Residents avoided engaging in conversations about health and avoided linking their health to their vulnerability to housing displacement pressure.	Data collection occurring during the COVID-19 pandemic, an additional stressor, likely impacted participants' perspectives<br><br>Participants who lacked documentation experienced constrained opportunities on the rental market, which may have impacted the data on their experience of exclusionary displacement pressure.<br><br>Author noted that as a White woman researcher, her social positionality may have influenced the participants approach to expressing their perspectives

Citation	Purpose	Sample & Setting	Design & analytic strategy	Definition of Structural vulnerability	Operationalization of Structural Vulnerability	Health Outcomes/Related Factors	Key Findings	Limitations
Worby et al. (2014)	To explore how Latino migrant day laborers distinguish problem drinking from other kinds of drinking, and what actions they take in response to problem drinking.	51 Latino migrant day laborers (LMDLs)<br><br>Primarily from Mexico and Guatemala<br><br>Mean age = 38 years<br><br>Mean age at first migration = 28 years (range 13 – 49)<br><br>Mean years in United States = 10 years (range: <1 – 35)<br><br>San Francisco and Berkeley, CA<br><br>Year unspecified	Ethnography and in-depth semi-structured interviews<br><br>Data collected over one year via naturalistic and participant observations and in-depth semi-structured interviews<br><br>Part of a larger National Institute on Alcohol Abuse and Alcoholism research project to develop and test a structural-environmental model of alcohol-related sexual HIV risk and prevention among LMDLs.<br><br>Thematic analysis	"Structural vulnerability refers to the social positionality of LMDLs that have migrated to the United States, a population characterized by exposure to harsh living and working conditions, lack of documentation, and characteristics produced and reproduced by global economic, political, social, and cultural factors."	A guiding framework, adapted from structural approaches applied in HIV research such as the continuum of causality beginning with distal structural and environmental factors, leading to proximal situational and individual level factors.<br>The focus on structural vulnerability especially highlights how structural factors shape specific patterns of social suffering.	Problem drinking	Many day laborers drink to forget their migration-related troubles, especially those prompted by being far from home and family and because of economic difficulties, and frequent unemployment, which appear to increase risk of problem drinking<br>The stressful and limited circumstances of LMDLs combined with what men described as the wide availability and relative affordability of liquor in the U.S. combine to push recreational drinking into problem alcohol use.<br><br>Although some day laborers work hard to avoid drinking situations, lack of financial resources, limited mobility, and restricted social networks limit their ability to carry out these strategies. Escaping influences to drink would depend on having access to affordable places to live that do not include being surrounded by substance users.	Social desirability bias related to alcohol use may have impacted the participants' self-report of problem drinking.

Citation	Purpose	Sample & Setting	Design & analytic strategy	Definition of Structural vulnerability	Operationalization of Structural Vulnerability	Health Outcomes/Related Factors	Key Findings	Limitations
Yang et al. (2014)	To understand Chinese immigrants' experiences with mental illness stigma and mental health disparities and to identify how the interaction between structural discrimination and cultural engagements might shape stigma.	50 Chinese immigrants<br><br>Mean age = 35 years<br><br>62% male<br>38% female<br><br>22% undocumented<br><br>Majority were diagnosed with schizophrenia or schizoaffective disorder<br><br>Two Chinese bilingual psychiatric inpatient units<br><br>New York City, NY<br><br>2006 - 2010	Semi-structured interviews<br><br>Data collected from narratives on stigma generated by the Chinese immigrant individuals with mental illness from a larger study funded by the National Institutes of Mental Health<br><br>Directed content analysis	"Structural vulnerability encompasses the political and institutional forces that comprise structural discrimination and structural violence, and focuses on individual 'positionality' or the vulnerability created by location within hierarchical relationships of power."	Structural vulnerability, operationalized as a theoretical framework, integrated with the 'moral experience' framework to conceptualize the interaction between structural mechanisms and local cultural processes that produce pronounced mental health disparities among socioeconomically deprived new immigrants.<br><br>*Moral experience framework considers aspects of culture as integral to the production of "local moral worlds" – social life that revolves around the pursuit or preservation of fundamental lived values that "matter most."	Mental health<br>Mental illness stigma	Study participants sought to work to accrue material wealth social status, and gain legal status, within the constraints of their structural vulnerability<br><br>Structural discrimination made mental health treatment significantly more complex and costly for new immigrants to the US, reinforcing treatment nonadherence and their vulnerability to mental illness stigma<br><br>Internalized structural vulnerability perpetuated structural discrimination as participants described acting within their prescribed social position	Convenience sampling from Chinese specialty inpatient units biased towards study participants who have access to that level of care.

Citation	Purpose	Sample & Setting	Design & analytic strategy	Definition of Structural vulnerability	Operationalization of Structural Vulnerability	Health Outcomes/Related Factors	Key Findings	Limitations
Young et al. (2022)	To identify the structural factors that may be mechanisms between enforcement policy and immigrant health through the perspective of Latino immigrants.	14 respondents, Latino immigrants<br><br>Mean age = 40 years (range 19 - 59)<br><br>Mean years in US = 26 years (range 14 – 48)<br><br>50% Female<br><br>Predominantly from Mexico<br><br>Two southern California counties<br><br>2015	Unstructured, open-ended interviews<br><br>*Testimonios* a narrative methodology<br><br>Iterative analysis using *in vivo* codes	"Structural vulnerability situates immigrants within mutually reinforcing insults ranging from the economic and political to the cultural and psychodynamic."<br><br>"This theory explicitly acknowledges the risk environment constraining individuals."	Structural vulnerability theory applied as a lens to understand mechanisms between policy and health	Mental and physical health and general well-being of Latino immigrants	The enforcement system was perceived as a daily, interconnected experience of physical, legal, institutional, and economic exclusion.<br><br>Exclusions defined what it meant to be a Latino immigrant in the US – living in precarity with an individual responsibility for overcoming obstacles - deportation was the most severe form of physical exclusion.<br><br>Legal exclusions present barriers to obtaining rights, legal identity, and access to opportunities essential to social and economic mobility.<br><br>The policies and practices of institutions like schools, healthcare, social services, immigration, and law enforcement are exclusionary and foster an internalized fear of seeking access to services.<br><br>Respondents encountered discrimination in the workforce contributing to their economic insecurity.<br>Economic barriers limited their access to legal protections, and were internalized as personal responsibility, causing a strain in family structures.	Health status or outcomes received minimal explicit discussion.

Citation	Purpose	Sample & Setting	Design & analytic strategy	Definition of Structural vulnerability	Operationalization of Structural Vulnerability	Health Outcomes/Related Factors	Key Findings	Limitations
Zhen-Duan et al. (2022)	To understand how the COVID-19 pandemic impacted low-income individuals with SUD and how people adjusted to SUD treatment changes during "stay-at-home" orders in NYC.	20 adult participants, enrolled in Medicaid, receiving outpatient addiction treatment. e<br><br>Mean age = 52 years<br><br>16 Male<br>4 Female<br><br>All participants identified as being a racial/ethnic minority.<br><br>Most participants were receiving outpatient treatment for opioid use disorder or alcohol use disorder<br><br>New York City, NY<br><br>April 2020 and June 2020	Semi-structured, in-depth phone interviews<br><br>Thematic analysis	"Structural vulnerability and structural competency frameworks theorize that structural forces contribute to health inequities through attributions and assumptions that organize people within a social hierarchy, rather than situating health outcomes solely within individuals' behaviors."	Structural vulnerability is operationalized as a framework, to assess the domains of social forces for these patients during the peak of the COVID-19 pandemic.<br><br>Interview guides targeted: the mental, physical, economic, and social impact of COVID-19 and the related changes in SUD treatment since the start of the pandemic.<br><br>Authors utilized a structural vulnerability framework "to conduct an inductive, thematic analysis on the combined audio-recordings, transcripts, and memos."	Substance use disorder treatment	Three themes resulted from the thematic analysis and corresponded with domains of structural vulnerability:<br>COVID-19 heightened financial instability increasing risks of COVID-19 infection<br><br>Stay-at-home orders limited access to resources but had positive impacts in strengthening social relationships and reducing substance use triggers<br><br>COVID-19 created challenges for treatment, however most described that SUD care improved during the pandemic<br><br>Increasing accessibility to care emerged as a potential structural level intervention for people who rely on SUD care.	Domains of SV are referenced but not explicitly identified<br><br>Purposeful and snowball sampling limited participants to those receiving outpatient care.<br><br>Phone interviews and may have excluded those without phone access
