# Supplementary material for: Structural Vulnerability in Health Research: A Systematic Mixed Studies Review
Source: J Adv Nurs. 2025 Dec 1;82(7):7104–25. doi: 10.1111/jan.70408 (PMC13267471; doi:10.1111/jan.70408)
Supplement: Supplementary file 3 — Table S3: jan70408‐sup‐0003‐TableS3.pdf. [file JAN-82-7104-s001.pdf]

Supplementary Table 3: Indicators used to measure structural vulnerability in quantitative analysis

**Structural vulnerability Indicator**	**Descriptions of Indicators**	**Number of studies that incorporated the Indicator**
Housing & Living conditions	Past or current homelessness<br>Residential transience<br>Interpersonal factors associated with living conditions (including feeling safe where one lives)	17
Food insecurity	Hunger over the past week; going to bed hungry	12
Experiences with the criminal justice system	Ever in jail<br>History of arrest<br>History of incarceration	8
Educational attainment	Limited education<br>Earned a GED<br>Completed some college	8
Financial status/Income sources	Financial insecurity (debt, behind in rent, needing to borrow money)<br>Depending on someone for money<br>No monthly savings<br>Illegal income sources	7
Social services & social support	Obtaining syringes from a syringe service program;<br>Receipt of any public benefit (e.g. crisis intervention, food assistance, legal assistance)	6
Employment & working conditions	Working conditions<br>Employment status (Fulltime, part-time or unemployed)<br>History of sex work<br>Proportion of days/hours worked per week.	5
Health status and healthcare access	Covered by health insurance<br>Number of ED visits<br>Not visiting the doctor in the past 12 months<br>HIV status<br>History of substance use	4
Exposure to violence or abuse	Child abuse (physical or sexual)<br>Intimate partner violence (physical or sexual)<br>Client perpetrated violence (physical or sexual)	3
Discrimination	Experiences of discrimination<br>(Racial discrimination;<br>discrimination on the basis of sexuality)	2
